# A deep-learning framework for multi-level peptide–protein interaction prediction

**DOI:** 10.1038/s41467-021-25772-4

**Published:** 2021-09-15

**Authors:** Yipin Lei, Shuya Li, Ziyi Liu, Fangping Wan, Tingzhong Tian, Shao Li, Dan Zhao, Jianyang Zeng

**Affiliations:** 1 Institute for Interdisciplinary Information Sciences, Tsinghua University, Beijing, 100084 China; 2 Machine Learning Department, Silexon AI Technology Co., Ltd., Nanjing, China; 3 Institute of TCM-X, MOE Key Laboratory of Bioinformatics, Bioinformatics Division, BNRist, Department of Automation, Tsinghua University, Beijing, 100084 China

**Keywords:** Machine learning, Virtual drug screening

## Abstract

Peptide-protein interactions are involved in various fundamental cellular functions and their identification is crucial for designing efficacious peptide therapeutics. Recently, a number of computational methods have been developed to predict peptide-protein interactions. However, most of the existing prediction approaches heavily depend on high-resolution structure data. Here, we present a deep learning framework for multi-level peptide-protein interaction prediction, called CAMP, including binary peptide-protein interaction prediction and corresponding peptide binding residue identification. Comprehensive evaluation demonstrated that CAMP can successfully capture the binary interactions between peptides and proteins and identify the binding residues along the peptides involved in the interactions. In addition, CAMP outperformed other state-of-the-art methods on binary peptide-protein interaction prediction. CAMP can serve as a useful tool in peptide-protein interaction prediction and identification of important binding residues in the peptides, which can thus facilitate the peptide drug discovery process.

## Abstract (web summary)

Peptide-protein interactions play fundamental roles in cellular processes and are crucial for designing peptide therapeutics. Here, the authors present a deep learning framework for simultaneously predicting peptide-protein interactions and identifying peptide binding residues involved in the interactions.

## Introduction

Peptides play crucial roles in human physiology by interacting with a variety of proteins and participating in many cellular processes, such as programmed cell death, gene expression regulation, and signal transduction^1,2^. Owing to their safety, favorable tolerability profiles in human bodies, and good balance between flexibility and conformational rigidity, peptides have become good starting points for the design of novel therapeutics, and identifying accurate peptide–protein interactions (PepPIs) is crucial for the invention of such therapeutics. Despite this fact, it is generally time-consuming and costly to determine pepPIs experimentally^1,3^. To mitigate this issue, a number of computational methods have been developed to facilitate peptide drug discovery.

Sequence-based methods and structure-based methods are two mainstream approaches for protein–ligand interaction prediction. Sequence-based methods mainly exploit primary sequence information to model the interactions. For example, CGKronRLS^4^ and NRLMF^5^ calculate sequence similarities and then use machine-learning models to predict interactions between proteins and their ligands. These methods often require known protein–ligand interactions as supervised labels and pairwise similarity scores of proteins (or ligands) as input features, which is often impractical for large-scale data owing to the huge computational complexity of similarity calculation. In addition, these approaches are not able to identify crucial binding residues, which hits a roadblock in deciphering the underlying mechanisms of PepPIs. Structure-based methods such as molecular docking inherently tackle the problem by modeling structural poses at atom level and predicting binding affinities. There are many well-established docking strategies for determining PepPIs, which can be roughly divided into local (e.g., DynaRock^6^ and Rosseta FlexPepDock^7^) and global docking methods (e.g., PIPER-FlexPepDock^8^ and HPEPDOCK^9^) according to the extent of input structural information. Most of these docking approaches require three-dimensional (3D) structure information to calculate binding free energies. Unfortunately, solving such 3D structures is generally time-consuming and expensive^1^, letting alone consuming a large number of computational resources due to the high computational complexity of the energy functions.

More recently, the booming deep-learning technologies have provided feasible solutions to model protein–ligand or protein–protein interactions (PPI) with better accuracy while requiring less computational resources. For instance, Cunningham et al.^10^ developed a hierarchical statistical mechanical modeling (HSM) approach to predict the interactions between peptides and protein binding domains (PBDs). Wan et al.^11^ developed DeepCPI, a powerful computational framework that combines representation learning with a multimodal neural network to predict compound–protein interactions (CPIs), and Chen et al.^12^ presented a siamese residual recurrent convolutional neural network to predict PPIs.

Although the peptide drugs have increasingly attracted immense attention and the number of approved peptide therapeutics has been on the incline over the recent decades, only a few works have been proposed to exploit machine-learning or deep-learning methods to model pepPIs. Furthermore, for deciphering the underlying mechanisms of pepPIs, the existing approaches mainly focus on identifying peptide-binding residues on the protein surface, such as the sequence-based method PepBind^13^ and the structure-based method InterPep^14^. PepBind^13^ is a sequence-based method for peptide-binding residue prediction, which assumes that a protein would have fixed binding residues even interacting with different peptides. However, in many cellular processes, different peptides with diverse biological functions may present distinct binding poses to a single protein, which thus may involve different protein residues in the interaction. Therefore, PepBind intrinsically fails to model the situations that multiple peptides interacted with different regions of a protein surface^13^. InterPep combines a random forest model with hierarchical clustering to predict the regions of a protein structure where the input peptide is most likely to bind^14^, which requires a target protein structure and a peptide sequence, and thus its application may be limited to only those proteins with available 3D structural data.

Moreover, most of the existing computational methods in modeling pepPIs fail to answer an important question, which is frequently raised by pharmacologists–how to determine the contribution of each individual peptide residue to the binding activity? Therefore, there is a manifest need for addressing the following challenges: (1) identifying the pepPIs accurately and efficiently, taking account of information from both peptides and proteins; (2) possessing the great generalization ability to large datasets; and (3) detecting crucial binding residues of peptides that can provide useful hints for a downstream amino-acid substitution or backbone modification.

Inspired by the above observations, we propose CAMP, a deep-learning framework for simultaneously predicting pepPIs and identifying the binding residues along with the peptide sequences. We first construct comprehensive feature profiles of peptides and proteins based on their primary sequences, including secondary structures, hydrophobic, hydrophilic, and polar properties, intrinsic disorder tendencies, and the evolutionary information derived by sequence alignment^15–20^. Next, we design a multi-channel feature extractor to learn the latent information from these physicochemical and biochemical profiles. CAMP further exploits convolution neural networks (CNNs) and self-attention mechanisms to fully extract both local and global information to predict the binary interactions of the input peptide–protein pair and identify the binding residues along the input peptide sequence. The rich and multi-level supervision information enables CAMP to accurately predict pepPIs only based on sequence-based input information. Through comprehensive evaluation on several benchmark datasets and an independent test data set from the RCSB Protein Data Bank (PDB)^21,22^ and DrugBank^23–27^, we demonstrated that CAMP significantly outperformed other state-of-the-art methods on pepPI prediction and was able to accurately identify peptide-binding residues. We also examined the capability of CAMP in addressing three related tasks–peptide–PBD (protein binding domain) interaction prediction, peptide–protein affinity assessment, and peptide virtual screening, and further showed that CAMP achieved better performance than baseline methods in addressing these tasks. Overall, CAMP can provide a useful tool for predicting and deciphering pepPIs using only sequence-based information as input.

## Results

### Overview of CAMP

**Fig. 1:**
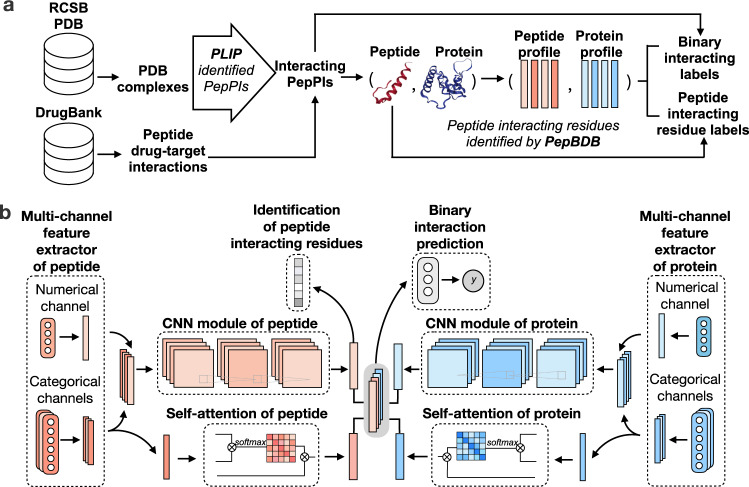
The workflow and architecture of CAMP. **a** Workflow of data curation and label extraction. We first extracted all PDB complexes containing peptides as ligands from the RCSB PDB^21,22^ and all peptide drugs with corresponding targets from DrugBank^23–26^. Then for the peptide–protein pairs from the PDB, we used PLIP^28^ to identify the interacting pairs by detecting whether there existed non-covalent interactions between them. Next, we generated sequence-based feature profiles for peptides and proteins, including residue-level structural and physicochemical properties, intrinsic disorder tendencies of peptides and proteins, and protein evolutionary information. We also downloaded the corresponding labels of peptide-binding residues from PepBDB^29^. Such residue-level labels and pairwise binary interactions were regarded as the multi-level supervised information for CAMP. **b** Network architecture of CAMP. Given the peptide feature profiles and the protein profiles of an input pair, the numerical features, i.e., the evolutionary protein PSSM and the intrinsic disorder tendency of each residue in the peptide or protein sequence are processed by the numerical channels of the feature extractors. The categorical features, i.e., the raw amino acids, secondary structures, polarity, and hydropathy properties of the peptide or protein are processed by three categorical channels. Next, the outputs of these channels are concatenated together and then fed into CNN modules, and the outputs of the amino-acid representations of the peptide and the protein are also fed into self-attention modules to learn the importance of individual residues (i.e., the contributions of individual residues to the final prediction). After that, the outputs of self-attention modules and CNN modules are concatenated together to predict a binding score for each peptide–protein pair through three fully connected layers and a binding score for each residue from the peptide sequence using the output of the CNN module of the peptide.

CAMP first applied the following five steps of multi-source data curation and multi-level label construction (Fig. 1a, more details can be found in Methods and Supplementary Note 10): (1) extracting peptide–protein complex structures from the RCSB PDB^21,22^ and the known drug-target pairs from DrugBank^23–27^; (2) using the protein–ligand interaction predictor (PLIP)^28^ to recognize non-covalent interactions between the peptide and the protein in each PDB complex, and only keeping the peptide–protein pairs with non-covalent interactions as positive samples; (3) deriving binding residue labels of the peptide from PepBDB^29^, a structure database of peptide–protein complexes derived from the RCSB PDB^21,22^; (4) generating residue-level structural and physicochemical properties, intrinsic disorder tendencies of peptides and proteins and protein evolutionary information based on the primary sequences of peptides and proteins; and (5) integrating multi-level labels, i.e., the binary interaction labels and peptide-binding residue labels of peptide–protein pairs, for the training process.

Figure 1b shows the overall network architecture of CAMP. Given the feature profiles of the input peptide–protein pair, CAMP exploits two multi-channel feature extractors to process them separately. Each extractor contains a numerical channel and three categorical channels. The numerical channel is used to extract the pre-defined dense features (i.e., the protein Position-specific scoring matrice (PSSM) and the intrinsic disorder tendency of each residue in both protein and peptide sequences). Each categorical channel contains a self-learning word embedding layer^30^, which takes one of the categorical features of the input peptide or protein (i.e., the raw amino acids, secondary structures, polarity, and hydropathy properties). Here, we design such a multi-channel architecture because the input profiles contain multifaceted features of different scales, which may bring inconsistency if we only use a simple encoder. Next, CAMP exploits two convolutions neural network (CNN) modules that extract the hidden contextual features of peptides and proteins, respectively. In addition, CAMP adopts self-attention mechanisms to learn the long-dependencies between residues and the contributions of individual residues of proteins and peptides to the final interaction prediction. After that, CAMP combines all the extracted features and uses three fully connected layers to predict whether there exists an interaction between a given peptide–protein pair. Furthermore, CAMP takes the output of the peptide CNN module with a sigmoid activation function for each position to predict whether each peptide residue binds to the partner protein. In our problem, the binary interaction prediction is our fundamental task and we aim to solve this problem by providing multi-level supervised information. Here, the extra binding residue labels can not only provide additional information to boost the performance of our main task, but also bring new insights about the pepPI by identifying the critical residues along with the peptide.

### CAMP outperforms baseline methods in binary interaction prediction

The binary classification of pepPIs is the primary goal of CAMP. Here, we compared the classification performance of CAMP with that of other state-of-the-art baseline methods, including a similarity-based matrix factorization method called NRLMF^5^, a deep-learning-based model for PPI prediction called PIPR^12^, and a deep-learning-based model for CPI prediction called DeepDTA^31^. All the prediction methods were evaluated on a benchmark data set through cross-validation. The area under the receiver operating characteristics curve (AUC) and the area under the precision-recall curve (AUPR) were used to evaluate the performance of all models. In general, AUPR can provide a better metric to evaluate the prediction models on skewed data in a more informative way than AUC^32^. To help readers estimate the difficulty of our task, we also reported the performance of several machine-learning baseline methods in Supplementary Note 1.

**Fig. 2:**
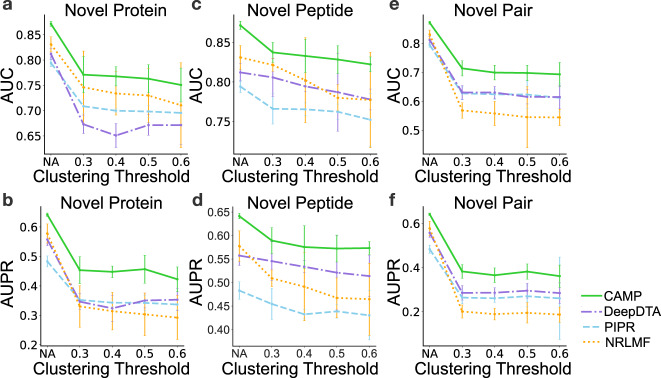
AUC and AUPR of CAMP and baseline models through cross-validation under three settings. **a**, **b** show the AUC and AUPR of CAMP and other baseline methods under the “novel protein setting”, respectively. **c**, **d** show the AUC and AUPR of CAMP and other baseline methods under the “novel peptide setting”, respectively. **e**, **f** show the AUC and AUPR of CAMP and other baseline methods under the “novel pair setting”, respectively. The error bars under “novel protein setting” and “novel peptide setting” represent the mean ± standard deviation over five folds (*n* = 5). The error bars under “novel pair setting” represent the mean ± standard deviation over nine folds (*n* = 9). “NA” stands for random cross-validation, i.e., randomly splitting the data set and used 80% of the data set to train the model and the remaining 20% to evaluate the performance.

Since the human-curated data may contain “redundant” interaction pairs (e.g., one protein interacting with more than one similar peptide or vice versa), which could be easily predicted by the models. To avoid the trivial predictions caused by such cases, we followed the same strategy as in MONN^33^, and mainly used the cluster-based cross-validation settings for performance evaluation. In particular, based on similarity scores derived from Smith-Waterman alignment (https://github.com/mengyao/Complete-Striped-Smith-Waterman-Library), we divided proteins and peptides into different clusters such that the entities from the same cluster did not appear in the training and testing sets at the same time (more details can be found in Supplementary Note 8). We evaluated the performance of CAMP and the baseline methods under three cluster-based cross-validation settings. More specifically, in the “novel protein setting”, no proteins from the same cluster appeared in both training and testing sets; in the “novel peptide setting”, no peptides from the same cluster appeared in both training and testing sets; and in the “novel pair setting”, neither proteins nor peptides from the same cluster appeared in training and testing sets at the same time. Figure 2 shows that CAMP consistently outperformed the state-of-the-art baseline methods, with an increase by up to 10% and 15% in terms of AUC and AUPR, respectively. In addition, we observed a slight decreasing trend of prediction performance for all methods with larger clustering thresholds, which generally corresponded to more difficult tasks. We also noticed that the model performance under the “novel peptide setting” seemed to be better than that in the other settings. This can be explained by the fact that the peptides in our benchmark set shared less similarity with each other than proteins, and thus the distributions of peptides in the training and testing sets did not change much after clustering based on similarities. Such test results suggested that CAMP can achieve better and more robust performance than the baseline methods under all cross-validation settings.

Figure 2 also shows that CAMP generated relatively variant prediction results under certain cluster settings. To further investigate the potential factors that cause this phenomenon, we conducted additional analyses using a fivefold cross-validation procedure on the binary prediction task (in Supplementary Note 1). Our analysis result (Supplementary Fig. 1) indicated that the relatively large prediction errors under two clustering settings may result from certain protein families, domains, and organisms (e.g., histone and GPCR for the protein families, trypsin and kringle for the domains, and bovine for the protein organisms).

Furthermore, we conducted comprehensive ablation studies to demonstrate the importance of individual components of CAMP, including different groups of features and the self-attention modules in the network architecture (Supplementary Note 2). Our ablation studies (Supplementary Table 2 and Supplementary Fig. 2) demonstrated that the current model architecture and feature selection scheme were optimal for our prediction task.

### New insights by characterizing binding residues on peptides

So far, a number of computational methods have been developed for predicting the interacting sites on the protein surface in PepPI predictions^14,34,35^. These methods learn from 3D structure information of peptide–protein complexes and can pinpoint interacting sites on protein surfaces with relatively good accuracy. However, few models are specifically designed to characterize interacting sites on the peptides in PepPIs, which are also crucial for understanding the biological roles of peptides and designing efficacious peptide drugs. For pharmacologists, the choice of chemical modification heavily relies on the identification of essential peptide residues involved in binding activities^1^. Conventionally, pharmacologists would iteratively replace possible residues and conducted wet experiments for verification. Although these attempts could provide useful information for further drug design, e.g., changing particular non-binding residues or modifying groups on their side chains to improve stability and reduce toxicity^1,2^, these experimental approaches are generally expensive and time-consuming.

**Fig. 3:**
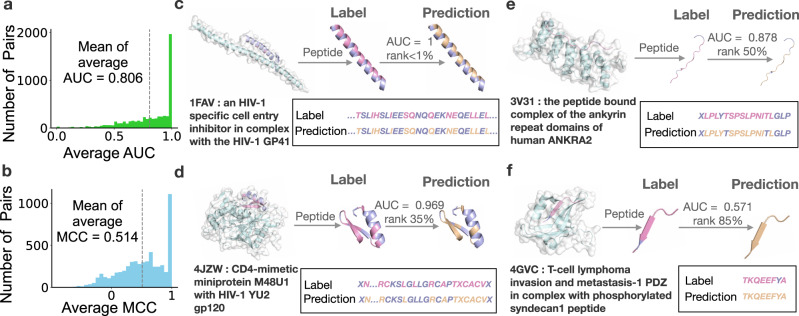
Performance evaluation of CAMP on peptide-binding residue identification on the benchmark data set through fivefold cross-validation. **a**, **b** show the distributions of AUC and MCC for peptide-binding residue prediction, respectively. The mean values of average AUC and MCC are plotted in dotted lines. **c**–**f** show four examples of peptide-binding residue identifications by CAMP that ranked ~1%, 35%, 50%, and 85% in terms of average AUC, respectively. The PDB complexes were retrieved from the RCSB PDB^21,22,59^ and the images were generated by PyMOL^60^. The protein chains in the complexes are colored in light blue while the peptide chains are colored in light purple and pink. For each peptide, the true binding residues are colored in pink while the predicted binding residues generated by CAMP are colored in wheat.

In CAMP, we designed a supervised prediction module to identify binding residues from a peptide sequence. We first constructed a set of qualified labels for peptide-binding residues using the interacting information derived from PepBDB^29^, which is a comprehensive structure database containing the known interacting peptide–protein complexes from the RCSB PDB^21,22^ and information about binding residues in peptides involved in hydrogen bonds and hydrophobic contacts. With the support from such supervised information, CAMP achieved an average AUC of 0.806 and Matthews Correlation Coefficient (MCC) (definitions can be found in Supplementary Note 9) of 0.514 on peptide-binding residue identification using a fivefold cross-validation procedure under the “random-split setting” (Fig. 3a, b). The cross-validation results under other settings can be found in Supplementary Note 3.

To further demonstrate the performance of CAMP in binding residue prediction, we also selected four representative cases (ranked ~1%, 35%, 50%, and 85% in terms of the average AUC scores of predicted peptide-binding residues, respectively) and compared the predicted residues with the true interacting ones. Figure 3c shows the first example, a complex of an HIV-1-specific cell entry inhibitor and HIV-1 GP41 trimeric core (PDB ID: 1FAV [10.2210/pdb1FAV/pdb]). The peptide inhibitor has 33 amino acids and 12 of them are binding residues. CAMP identified all these binding residues without any false positives. Such a prediction was the most ideal case in our prediction task and we found that 30.2% of the binding residue identification was completely accurate like this case. Figure 3d shows the second example, a complex of HIV-1 gp120 envelope glycoprotein and the CD4 receptor (PDB ID: 4JZW [10.2210/pdb4JZW/pdb]), which ranked around the top 35% in terms of the average AUC. The peptide has 28 amino acids and 13 of them are binding residues. Our predicted binding residues covered 11 true binding residues along the peptide sequence and missed two true binding residues. Figure 3e shows the third example, a complex of a peptide from histone deacetylase and the ankyrin repeat family A protein (PDB ID: 3V31 [10.2210/pdb3V31/pdb]). This pair ranked around the median among our predictions in terms of AUC and 11/13 of the true binding residues were successfully identified by CAMP with one false positive. Figure 3f shows the last example, a complex of the T-lymphoma invasion and metastasis inducing protein and an eight-residue phosphorylated syndecan-1 peptide (PDB ID: 4GVC [10.2210/pdb4GVC/pdb]), which ranked ~85% among our predictions with an average AUC of 0.571. All eight residues including one false positive were predicted as binding residues by CAMP. Overall, our test results demonstrated that CAMP yields accurate binding residue predictions and thus can provide reliable evidence for further understanding the interacting mechanisms of peptides with their partner proteins.

### Identifying GLP-1 receptor as a target of Semaglutide and its analogs

Glucagon-like peptide receptor (GLP-1R) agonists play an important role in the treatment of type 2 diabetes mellitus^36,37^. We next investigated whether CAMP was able to correctly identify the interactions of Semaglutide, a known GLP-1R agonist (GLP-1RA), and its analogs with GLP-1R. In our benchmark data set, there are seven Semaglutide-analogous peptides that bind to GLP-1R. To avoid “easy prediction”, we removed those GLP-1RA peptide drugs from the training set that shared similar sequences (defined as peptide sequence similarities >40%) with Semaglutide (e.g., Liraglutide and Taspoglutide), and had interacting proteins similar to GLP-1R (i.e., with protein sequence similarities >40%). After removing these records as well as seven pairs of Semaglutide-analogous peptides and GLP-1R, we re-trained the CAMP model and combined the seven Semaglutide-analogous peptides with the remaining 3400 proteins to construct an independent test set which contained 23,800 candidate pairs. The test showed that CAMP was able to identify six of seven interacting pairs of Semaglutide-analogs peptides and GLP-1R with an AUC score of 0.831. For all the Semaglutide-analogs peptides, GLP-1R was ranked to the top 10% almost among all the candidate proteins (more details can also be found in Supplementary Table 3 and Supplementary Fig. 7). Such results further demonstrated the strong predictive power of CAMP.

We also examined the predicted binding residues of Semaglutide with its receptor (detailed results can be found in Supplementary Fig. 8 and Supplementary Note 4). CAMP correctly identified 11/12 of the true binding residues of Semaglutide with an average AUC of 0.917. Such a prediction result can provide useful insights for pharmacologists if they aim to improve the stability of the peptide drugs by replacing the non-binding residues with synthetic amino acids without changing the interacting interface of the binding complexes.

### Generalizability of CAMP on additional benchmark datasets

We conducted additional tests to further illustrate the generalizability of CAMP on binary interaction prediction and peptide-binding residue identification. In particular, we first evaluated CAMP on an additional independent data set derived from the PDB^22,38^ following the same strategy as in constructing our previous benchmark data set. This additional test set contained 379 PepPIs from 262 peptides and 246 proteins from the PDB complexes released from 1 October 2019 to 10 March 2020. The corresponding PDB IDs and UniProt IDs can be found in Supplementary Tables 13 and 16 in Supplementary data. We also randomly paired these peptides and proteins without known evidence of interactions in the test set to obtain negative samples.

**Fig. 4:**
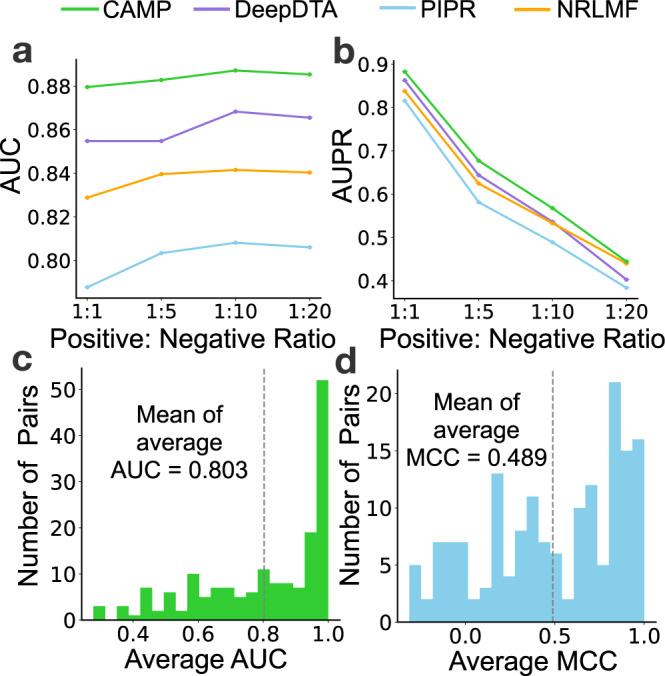
CAMP yielded robust performance and outperformed the baseline models on an independent test set. **a**, **b** show the evaluation results with different positive-negative ratios of the test data set in terms of AUC and AUPR, respectively. **c**, **d** show the distributions of AUC and MCC for peptide-binding residue prediction, respectively. The mean values of average AUC and MCC are plotted with dotted lines.

To demonstrate the robust performance of CAMP on binary interaction prediction, we evaluated the performances of CAMP and the baseline models on several variations of the above test data set with different positive-negative ratios. Each model was first trained on the complete benchmark data set and then an ensemble version (i.e., average predictions from five models) was used to make predictions on the additional test datasets. Figure 4a and b show that CAMP achieved the best results under all scenarios, demonstrating that CAMP outperformed the baseline methods with a relatively robust performance. We also observed that the AUC of all methods increased slightly as the positive-negative ratio decreased from 1:1 to 1:10. This was probably because the increased sample size brought more information for models to learn. Also, the AUPR of all methods decreased more dramatically than AUC as the positive vs. negative ratio increased. This was mainly because AUPR is generally more affected by the ratio of positive vs negative samples^32^.

We also evaluated the prediction results of CAMP on the identification of peptide-binding residues. We obtained the annotated binding residues of peptide sequences from PepBDB^29^. In total, 208 PepPIs have such peptide-binding residue labels from the test data set. Figure 4c and d show that CAMP was able to maintain its prediction power on the above additional data set.

We additionally compared CAMP with other methods on several representative benchmark data sets (Supplementary Table 4) that were originally used to evaluate the performance of peptide docking and detecting “hotspots” at protein interface^34,39–42^. As shown in Supplementary Fig. 9, CAMP still outperformed the baseline methods on all these additional datasets in terms of both AUC and AUPR scores. These additional evaluation results further demonstrated the superior predictive power and generalizing ability of CAMP.

### Extended applications of CAMP in three related tasks

We further investigated the application potential of CAMP in three related tasks, i.e., predicting peptide–PBD (protein binding domain) interaction prediction, binding affinity assessment, and virtual screening of peptides. For predicting peptide–PBD interactions, although we rarely found deep-learning-based methods for predicting PepPIs, there was a machine-learning approach, called HSM^10^, focusing on a quite related problem, i.e., predicting the interactions between peptides and globular PBDs. The PBD-containing proteins play essential roles in a variety of cell activities, e.g., multiprotein scaffold formation and enzyme activity regulation^38,43,44^. By incorporating biophysical knowledge as prior information into a machine-learning framework, HSM was reported to yield superior prediction performance on eight common PBD families with AUC scores ranging from 0.88 to 0.92. We compared CAMP with two reported models of HSM, i.e., HSM-ID (in which eight separate models were trained for each PBD/enzyme family) and HSM-D (in which a single unified model was trained for all families), on predicting peptide–PBD interactions. Here, we compared the performance of CAMP with that of HSM models on predicting peptide–PBD interactions. In particular, we evaluated the performance of CAMP with the same data set and eightfold cross-validation setting as used in the HSM paper (see Supplementary Note 6 for more details).

**Fig. 5:**
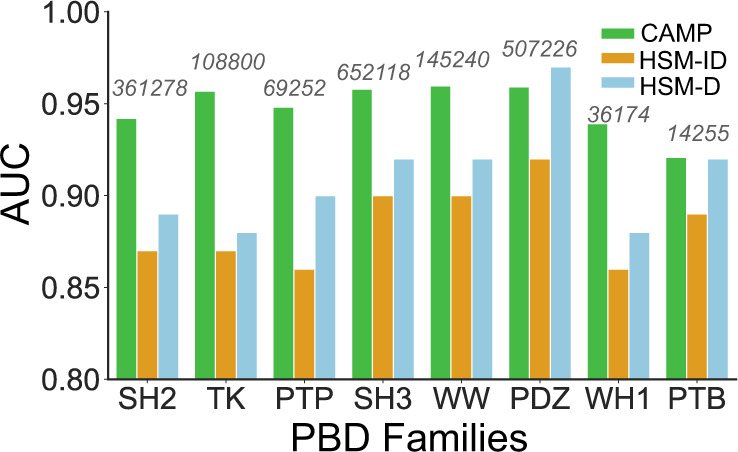
Model performance of CAMP, HSM-ID, and HSM-D across eight families. CAMP achieved a relatively stable performance overall families, whereas the performances of HSM models were easily influenced by the sample size (marked in gray number) of the training set. CAMP outperformed the HSM models, with an increase of AUC by 3–7%. All the evaluation metrics of the HSM models were obtained from the origin paper^10^.

Figure 5 shows that CAMP significantly outperformed both HSM-ID and HSM-D across all domain families except the PDZ family. We also noticed that HSM-ID and HSM-D had large prediction variations across different families. As explained in the HSM paper, this may be due to the skewed distribution of the data (i.e., the numbers of pairs from different families were imbalanced). For families of large data amounts like PDZ, the HSM models could learn quite well but for those families of relatively small data sizes like domains from the phosphotyrosine binding family, HSM models had an obvious drop in performance. In contrast, the performance of CAMP was more robust and less influenced by the fluctuant data sizes. Such results indicated that CAMP is also suitable for tackling the related peptide–PBD interaction prediction problem.

Next, we investigated whether CAMP can also be applied to assess the binding affinity of peptide–protein pairs. Here, we made a comparison between CAMP and several baseline methods, including random forest (a conventional machine-learning based framework), DeepDTA (a deep-learning-based framework)^31^, and AutoDock CrankPep (a structure-based docking method)^45^, on an affinity data set derived from PDBbind v2019^46^ (more details about data processing can be found in Supplementary Note 6). As shown in Supplementary Table 5, CAMP achieved higher performance than all the baseline methods with higher Pearson correlation coefficients and smaller prediction errors in terms of RMSE. Considering that CAMP was not particular designed for affinity prediction and the limited size of training data, such a comparison result was satisfactory and further illustrated the great potential of CAMP in predicting binding affinities between peptides and proteins. We also investigated whether CAMP can be applied for virtual “alanine scanning”, as the experimental “alanine scanning” strategy is considered as a “standard” in affinity assessment. Since there was no public data that can comprehensively cover the experimental “alanine scanning” affinities for all protein–peptide complex structures available from the RCSB PDB^21,22^, here we only chose two peptide–protein complexes (PDB IDs: 4TMP [10.2210/pdb4TMP/pdb], 4N4H [10.2210/pdb4N4H/pdb]) as case studies instead of performing a systematic evaluation (more details can be found in Supplementary Note 6). As shown in Supplementary Fig. 10, the Pearson correlation coefficients between the logarithms of experimental affinities and the prediction scores were 0.6284 and 0.5646, for the PDB complexes 4TMP and 4N4H, respectively, which indicated that CAMP can capture the variation tendency of binding affinities in the “alanine scanning” experiments to a certain degree. In a real application scenario, CAMP can be used to rank the virtual “alanine scanning” results to determine which residues are more important for the binding activities.

Furthermore, we evaluated the capability of CAMP and various docking methods, including CABS-Dock^47^, MDockPeP^48^, AutoDock CrankPep v1.0^45^, and GalaxyPepDock^49^, for virtual screening of peptides (Supplementary Note 6). We observed that CAMP achieved better performance than those structure-based docking methods (Supplementary Table 6). It was not surprising to observe such comparison results because these structure-based docking methods were originally designed for binding pose prediction rather than virtual screening. Considering the above fact, we believe that CAMP can provide a more suitable and powerful tool than those structure-based docking methods on the virtual screening of peptides.

## Discussion

In this work, we proposed CAMP, a deep-learning framework for multi-level peptide–protein interaction prediction, including binary interaction prediction and peptide-binding residue prediction. We first generated a series of sequence-based features to construct feature profiles for peptides and proteins. Compared with traditional peptide or protein feature representations such as k-mer, our comprehensive feature profiles combined informative structurally annotated features, evolutionary information, and intrinsic disorder tendency scores to enhance the peptide–protein interaction prediction. We then used multi-channel feature extractors to separately process numerical and categorical features to avoid the inconsistency of multi-source features. Comprehensive cross-validation evaluation demonstrated the superior performance of CAMP over the state-of-the-art baseline methods on binary interaction prediction. Furthermore, we sought to decipher the underlying mechanisms of peptide–protein interactions by identifying the peptide-binding residues. We showed that CAMP can accurately detect the binding residues from the peptide sequence. We also presented four representative cases to visualize the results of the peptide-binding residue identification task and examined the predicted targets for Semaglutide and its analogs. We also verified the application potential of CAMP in peptide–PBD interaction prediction, binding affinity assessment of peptide–protein pairs, and virtual screening of peptides. All these results indicated that CAMP can provide accurate peptide–protein interaction predictions as well as useful insights into understanding the peptide-binding mechanisms.

Comparing with structure-based docking methods, CAMP offers various advantages. For example, CAMP can simultaneously fulfill the tasks of predicting binary interactions and identifying the peptide-binding residues involved in the interactions, whereas previous structure-based methods only focus on predicting the binding poses or identifying the binding regions at the protein surface. In addition, for a single peptide–protein pair, CAMP makes the prediction in seconds while the structure-based docking methods usually take hours. Furthermore, CAMP only requires sequence information as input, and thus does not rely on the limited structure data. More specifically, there are 564,638 proteins with manually annotated sequence information in the Swiss-Prot database^50^, but only 8.49% of them have the solved structures. Under such a circumstance, CAMP is able to make predictions for much more target proteins than the current structure-based methods and thus will have a much wider range of applications.

Nevertheless, there still exist certain limitations in the current version of CAMP. For example, it cannot directly predict the binding residues from the protein sequence in a given peptide–protein pair. In fact, we had explored whether CAMP can predict the binding residues of proteins. Under the “random-split setting” of fivefold cross-validation, when adding a module of predicting protein-binding residues, CAMP identified fewer than 20% of real binding residues and the average AUC of the binary interaction prediction task slightly decreased to 0.843. The relatively unsatisfied result on the protein-binding residue prediction in our framework was probably due to the following challenges. First, the protein sequences are generally much longer than the peptides, ranging from 52 to 4911 residues, posing difficulty in pinning down the exact interacting residues. Second, certain uncertainty may arise when extracting the positive labels of protein-binding residues from co-crystal complex structures using PLIP. In the future, we are planning to incorporate more data such as binding domain information to further improve the results on predicting binding residues in the proteins.

## Methods

### Data sets

We constructed a benchmark data set from two sources, i.e., protein–peptide complex structures from the RCSB PDB^21,22^ and the known drug-target pairs from DrugBank^23–27^ (more details of data curation can be found in Supplementary Note 10 and the corresponding PDB IDs that we used for training and testing can be found in Supplementary Tables 12 and 13 in Supplementary Data, respectively. The DrugBank IDs that we used can be found in Supplementary Table 14 in Supplementary Data). In total, we obtained 7417 positive interacting pairs covering 3412 protein sequences and 5399 peptide sequences. Among them, 6581 pairs from the RCSB PDB have residue-level binding labels in peptide sequences. We then constructed a negative data set by randomly shuffling those non-interacting pairs of proteins and peptides. More specifically, for each positive interaction, five negatives were generated by randomly sampling from all the shuffled pairs of non-interacting proteins and peptides. Overall, we obtained 44,502 peptide–protein pairs as our benchmark data set.

### Problem formulation

In our problem setting, we mainly considered the lengths of peptide sequences ≤50, and the lengths of protein sequences longer than 50. Peptides with fewer than 50 residues were zero-padded to have the same input feature length (more details can be found in Supplementary Note 10). We use ${{{{{{{\mathcal{A}}}}}}}}$ to denote a vocabulary of 21 types of amino acids (i.e., 20 canonical amino acids and a letter “X” for any unknown or non-standard amino acid). Then, a given peptide–protein pair (**S**_**pep**_, **S**_**pro**_) can be defined as two sequences of amino acids **S**_**pep**_ = (*p*_1_, *p*_2_, ..., *p*_*m*_), **S**_**pro**_ = (*q*_1_, *q*_2_, ..., *q*_*n*_), in which each ${p}_{i},{q}_{j}\in {{{{{{{\mathcal{A}}}}}}}}$ stand for the residue at position *i* of the peptide and position *j* of the protein, respectively, and *m*, *n* represent the lengths of the peptide and protein sequences, respectively.

Our sequence-based neural network model, CAMP, addresses two prediction tasks: (1) a binary classification task to predict PepPIs; (2) a binding residue classification task to identify interacting sites from the input peptide sequence. More specifically, the first prediction task can be described as a binary classification problem, in which label *y*_*i*_ = 1 indicates the existence of an interaction between the *i*th peptide–protein pair and *y*_*i*_ = 0 otherwise. The output probability of CAMP for this task can be denoted by a real value between 0 and 1. The second prediction task aims to pinpoint the binding residues from the peptide sequence in a given peptide–protein pair. Here, for a peptide with *m* residues, we define its binding vector as **b**_**pep**_ = (*b*_1_, *b*_2_, ..., *b*_*m*_), in which each binary element *b*_*i*_ denotes whether the *i*th residue binds to the partner protein (1 for the existence of binding and 0 otherwise).

### Construction of sequence-based feature profiles

CAMP only requires raw sequences to construct the feature profiles of peptides and proteins, therefore alleviating the problem of limited structure data. In particular, CAMP incorporates multifaceted features, including the structure-based and physicochemical properties of individual residues in peptide and protein sequences, protein evolutionary information, and the disorder tendencies of peptide and protein sequences.

#### Residue-level structural and physicochemical properties

We first define an alphabet of 21 elements to describe different types of amino acids (i.e., 20 canonical amino acids and a letter “X” for unknown or non-standard ones). Each type of amino acid is encoded with an integer between 1 and 21. For each amino-acid sequence **S** = (*a*_1_, *a*_2_, ..., *a*_*n*_), we generate an *n* × 1 array, in which in the corresponding residue position, each element is an integer representing the amino-acid type.

In addition, although our problem setting assumes that 3D structure data are unavailable, previous studies have suggested that the predicted structures of the amino-acid sequences could still provide useful information^16,51,52^. Here, for each amino-acid sequence **S** = (*a*_1_, *a*_2_, ...., *a*_*n*_), we use SSPro^16^ to generate an *n* × 1 array, in which each element is an integer representing the combination of secondary structure class and amino-acid type at the corresponding position (see Supplementary Note 7).

Furthermore, the hydrophobicity, hydrophilicity, and polarity of the R groups of individual amino acids can affect the tendency of the interactions between residues^53^. For each amino-acid sequence **S** = (*a*_1_, *a*_2_, ..., *a*_*n*_), we generate an *n* × 1 array, in which each element is an integer representing the combination of the polarity and hydropathy properties of the residue at the corresponding position (see Supplementary Note 7).

#### Protein evolutionary information

PSSMs are popular representations of protein sequences, which can detect remote homology of the protein sequences^20,54^. For each protein sequence **S** = (*a*_1_, *a*_2_, ..., *a*_*n*_) of length *n*, we use PSI-BLAST^19^ to generate a normalized position-specific scoring matrix, an *n* × 20 array **S**, in which each element *S*_*i*,*j*_ stands for the probability of the *j*th amino-acid type at position *i* in the protein sequence (see Supplementary Note 7).

#### Intrinsic disorder tendencies to form contacts

It has been reported that the intrinsic disorder-based features in peptide and protein sequences play a crucial role in protein–peptide interactions^15^. Here, for individual residues in the peptide and protein sequences, we first employ IUpred2A^17,18^ to predict its intrinsic disorder properties. For an amino-acid sequence **S** of length *m*, we construct an *m* × 3 arrays representing three types of disorder scores for individual residues (see Supplementary Note 7).

#### Multi-channel feature extractors

To avoid the inconsistent scales of different features within the profiles (i.e., the disorder and PSSM features are dense vectors while residue-level properties are categorical vectors), CAMP exploits two multi-channel feature extractors to derive the encoded features, which process the protein and peptide profiles separately. Each extractor has three categorical channels and one numerical channel (Fig. 1b). Each categorical channel consists of three self-learning word embedding layers^30^, taking amino acids, secondary structures, and physiochemical representations as input, respectively. Each numerical channel consists of a fully connected layer to take dense features as input, i.e., the intrinsic disorder tendencies features (ranging between 0 and 1) of peptides and proteins as well as the normalized evolutionary matrices (PSSM) of proteins. These numerical features are pre-defined and calculated based on primary sequences.

### Capturing the dependency relations between residues

Learning the local relations between neighboring residues and capturing long-range dependencies between residues from the whole sequences are key points in our task. CAMP exploits CNN modules and self-attention mechanisms to extract such latent information.

#### The CNN

We deploy a popular deep-learning architecture, CNN, to extract the informative knowledge from the input sequence-based features. The CNN architecture is able to integrate local dependencies to capture latent information of sequential features and has been successfully used to predict both PPIs and compound–protein interactions^31,33,55^. Here, we use two CNN modules to extract the hidden features of peptides and proteins separately. Each CNN module consists of three convolution layers with a rectified linear unit (ReLU) function followed by a max-pooling layer. The max-pooling layer down-samples the output of previous filters from convolution layers to learn the features for better generalization and also reduces the output of the ReLU layer to a one-dimensional array to achieve higher learning efficiency (see Supplementary Note 11 for more details).

#### Self-attention

We adopt a single-head self-attention mechanism in the CAMP framework, which has been widely used to capture long-range dependencies between tokens in sequential data^56^. More specifically, let ${{{{{{{\bf{U}}}}}}}}={\left\{{{{{{{{{\bf{u}}}}}}}}}_{i}\right\}}_{i = 1}^{N}$ denote the output vector of the embedding layer with basic amino-acid feature representation of an input sequence consisting of *N* residues, where **u**_**i**_ represents the *d*-dimensional embedded feature vector of the *i*th residue. Then, the output of a single-head self-attention module is a weighted sum of the feature vectors over all residues, that is,

1 $${g}_{i}=\mathop{\sum }\limits_{j=1}^{N}{{{{{\rm{softmax}}}}}}(\frac{{{{{{{{{\bf{q}}}}}}}}}_{i}{{{{{{{{{\bf{k}}}}}}}}}_{j}}^{T}}{\sqrt{{d}_{k}}}){{{{{{{{\bf{v}}}}}}}}}_{j},$$

where softmax( ⋅ ) stands for the softmax operation, ${g}_{i}\in {{\mathbb{R}}}^{{d}_{k}}$ stands for the output of the self-attention layer for **u**_*i*_ that implicitly indicates the response of features at the *i*th position, $\sqrt{{d}_{k}}$ stands for the scaling factor to control the magnitude of the dot product, and **q**_*i*_, **k**_*i*_ and **v**_*i*_ represent the query, key and value vectors of the *i*th residue, respectively, which are calculated by

2 $${{{{{{{{\bf{q}}}}}}}}}_{i}={{{{{{{{\bf{W}}}}}}}}}_{q}{{{{{{{{\bf{u}}}}}}}}}_{i},$$

3 $${{{{{{{{\bf{k}}}}}}}}}_{i}={{{{{{{{\bf{W}}}}}}}}}_{k}{{{{{{{{\bf{u}}}}}}}}}_{i},$$

4 $${{{{{{{{\bf{v}}}}}}}}}_{i}={{{{{{{{\bf{W}}}}}}}}}_{v}{{{{{{{{\bf{u}}}}}}}}}_{i},$$

where ${{{{{{{{\bf{W}}}}}}}}}_{q}\in {{\mathbb{R}}}^{{d}_{k}\times d}$, ${{{{{{{{\bf{W}}}}}}}}}_{k}\in {{\mathbb{R}}}^{{d}_{k}\times d}$, and ${{{{{{{{\bf{W}}}}}}}}}_{v}\in {{\mathbb{R}}}^{{d}_{k}\times d}$ stand for the learnt weight matrices of the query, key, and value vectors, respectively. This attention mechanism allows the model to focus on the crucial residues from the sequences dynamically and capture the contributions of features at individual residues to facilitate the final prediction.

### The multi-objective learning strategy

Here, we employ an idea of multi-objective training to simultaneously learn two tasks, i.e., the binary interaction prediction task and the peptide-binding residue identification task. The previously encoded features are fed into the two prediction modules separately and the losses of two tasks are optimized simultaneously during the training process.

#### The binary interaction prediction

CAMP aggregates the features from the CNN modules and the attention modules of peptides and proteins and fed them into the binary interaction prediction module, which consists of three fully connected layers. Each of the first two fully connected layers is followed by a dropout operation to alleviate the overfitting problem. We apply a sigmoid function $\sigma (x)=\frac{1}{1+{e}^{-x}}$ on the last layer to produce a final prediction, in which the prediction score ≥0.5 indicates that there is an interaction between the given peptide–protein pair, and <0.5 otherwise.

#### The peptide-binding residue prediction

Given a peptide–protein pair, we also design a prediction module to identify which residues from the peptide sequence bind to the protein partner. The output features **H** of the CNN module of the peptide can be denoted by its row vectors ${\left\{{{{{{{{{\bf{h}}}}}}}}}_{j}\right\}}_{j = 1}^{{N}_{k}}$, where each **h**_**j**_ stands for the feature vector of the residue at position *j* in the peptide. We apply a single-layer neural network on **h**_*j*_ and then normalized the output values using a sigmoid function to obtain a one-dimension value for each residue. Thus, the predicted score residue at position *j* in the peptide is

5 $${b}_{j}=\sigma ({{{{{{{{\bf{W}}}}}}}}}_{pep}{{{{{{{{\bf{h}}}}}}}}}_{j}+{c}_{j}),$$

where *j* = 1, 2, ..., *N*_*k*_, *N*_*k*_ represents the number of residues in the peptide sequence and *σ*(*x*) denotes the sigmoid function. Here, *b*_*j*_ ≥ 0.5 indicates that position *j* in the peptide is a binding residue, and *b*_*j*_ < 0.5 otherwise.

#### Dual-objective optimization

CAMP has two separate binary cross-entropy loss functions for the corresponding two classification tasks, i.e., the binary interaction prediction and the peptide-binding residue prediction. For the binary interaction prediction task, in a training set with *N* peptide–protein pairs, the binary cross-entropy loss is defined as

6 $${{{{{\rm{loss}}}}}}_{{{{{\rm{pair}}}}}}=-\frac{1}{N}\mathop{\sum }\limits_{i=1}^{N}{y}_{i}\cdot {{{{{{\mathrm{log}}}}}}}\,({y}_{i}^{\prime})+(1-{y}_{i})\cdot {{{{{{\mathrm{log}}}}}}}\,\left.(1-{y}_{i}^{\prime})\right),$$

where *y*_*i*_ and ${y}_{i}^{\prime}$ stand for the true binary label and the predicted interaction probability of a given peptide–protein pair, respectively.

For the peptide-binding residue prediction task, we also use a binary cross-entropy loss to measure the difference between predicted and real binding labels for individual residues in the peptide sequence. To ignore the padded zeros in our fixed-length input, we apply masks on those padded positions. More specifically, for the training set with *N* peptide–protein pairs, the masked cross-entropy loss for peptide-binding residue prediction is defined as

7 $${{{{{\rm{loss}}}}}}_{{{{{\rm{pep}}}}}}=-\frac{1}{N}\frac{1}{M}\mathop{\sum }\limits_{i=1}^{N}\mathop{\sum }\limits_{k=1}^{M}({b}_{ik}\cdot log({b}_{ik}^{\prime})+(1-{b}_{ik})\cdot log(1-{b}_{ik}^{\prime}))\cdot {m}_{ik},$$

where *m*_*ik*_ stands for the mask value at position *k* in the peptide sequence of sample *i* and *M*_*i*_ = ∑*m*_*ik*_ represents the number of residues in the padded peptide sequence of sample *i* (*m*_*ik*_ is 0 if position *k* is padded with zero and 1 otherwise), and *b*_*ik*_, ${b}_{ik}^{\prime}$ represent the true label and the predicted probability of position *k* in the *i*th sample, respectively.

The above two losses are combined together and optimized simultaneously in a multi-objective training process, that is,

8 $${{{{{\rm{loss}}}}}}_{{{{{\rm{total}}}}}}={{{{{\rm{loss}}}}}}_{{{{{\rm{pair}}}}}}+\lambda {{{{{\rm{loss}}}}}}_{{{{{\rm{pep}}}}}},$$

where *λ* stands for a weight parameter that balances the two losses. All parameters of CAMP are updated using the RMSProp optimizer^57^. The details about hyperparameter tuning and selection can be found in Supplementary Note 12. A single CAMP model can be trained within two hours on a linux server with 48 logical CPU cores and one Nvidia Geforce GTX 1080Ti GPU.

### Reporting summary

Further information on research design is available in the Nature Research Reporting Summary linked to this article.

## Supplementary information

Supplementary Information

Reporting Summary

## Data Availability

The peptide–protein complex structure data used in this study can be downloaded from the RCSB PDB database [https://www.rcsb.org/downloads/] and the structural peptide–protein interaction data with annotated binding residue information are available from PepBDB [http://huanglab.phys.hust.edu.cn/pepbdb/db/download/]. The corresponding PDB IDs that we used for training and testing the model can be found in Supplementary Tables 12 and 13 in Supplementary Data, respectively. The peptide drug-target interaction data are available from DrugBank [https://go.drugbank.com/releases/latest]. The sequence data of the peptide drugs on DrugBank are available from PubChem [https://pubchem.ncbi.nlm.nih.gov/]. The corresponding DrugBank IDs that we used can be found in Supplementary Table 14. The protein sequence data used in this study are available from UniProt [https://www.uniprot.org/downloads] and the corresponding UniProt IDs that we used for training and testing can be found in Supplementary Tables 15 and 16 in Supplementary Data, respectively. The peptide–PBD interaction data are available from [https://github.com/aqlaboratory/hsm]. The affinity data of peptide–protein interactions are available from PDBbind v2019 [http://www.pdbbind.org.cn/] and the corresponding PDB IDs that we used for affinity assessment can be found in Supplementary Table 17 in Supplementary Data. The supplementary test sets are available from LEADS-PEP [10.1021/acs.jcim.9b00905/suppl_file/ci9b00905_si_001.pdf], PPDbench [https://webs.iiitd.edu.in/raghava/ppdbench/dataset.php], PepSet [http://cadd.zju.edu.cn/pepset/], TS251 [https://bitbucket.org/isaakh94/interpep_pipeline/src/master/databases/] and TS125 [https://academic.oup.com/bioinformatics/article/34/3/477/4237510#supplementary-data], respectively. Source data are provided with this paper.

## Source data

Source Data

## Code availability

The source codes of CAMP are available on the GitHub repository at https://github.com/twopin/CAMP and Zenodo^58^.
