# Supplementary material for: A deep-learning framework for multi-level peptide–protein interaction prediction
Source: Nat Commun. 2021 Sep 15;12:5465. doi: 10.1038/s41467-021-25772-4 (PMC8443569; doi:10.1038/s41467-021-25772-4)
Supplement: Supplementary file 1 — Supplementary Information [file 41467_2021_25772_MOESM1_ESM.pdf]

Supplementary Information for "A deep learning framework for multi-level peptide-protein interaction prediction"

Yipin Lei [1], Shuya Li [2], Ziyi Liu [2], Fangping Wan [2], Tingzhong Tian [1], Shao Li [3], Dan Zhao [1,*], and Jianyang Zeng [1,*]

1 Institute for Interdisciplinary Information Sciences, Tsinghua University, Beijing 100084, China.

2 Machine Learning Department, Silexon AI Technology Co., Ltd., Nanjing, China.

3 Institute of TCM-X, MOE Key Laboratory of Bioinformatics, Bioinformatics Division, BNRIST, Department of Automation, Tsinghua University, Beijing 100084, China.

∗ All correspondence should be addressed to {zhaodan2018, zengjy321}@tsinghua.edu.cn.

Supplementary Note 1: Additional results on "CAMP outperforms baseline methods in binary interaction prediction"

To help readers evaluate the difficulty of our task, we also included the comparison results between our model and several machine learning baseline methods, including random forest, gradient boosting decision tree (GBDT), XGBoost and logistic regression. The input peptide and protein sequences were first zero-padded (i.e., using the same padding strategy as in CAMP) and each residue in the sequences was one-hot-encoded. Next, the feature representations of the peptide and the protein were concatenated together and used as input information. We determined the hyper-parameters of these machine learning baselines through five-fold cross-validation using the grid search strategy on the benchmark dataset. More details about the hyper-parameter determination for these machine learning methods can be found in Supplementary Note 12. Supplementary Table 1 shows the prediction performance of CAMP and these machine learning baseline methods under the "random-split setting", in terms of both AUC and AUPR scores. The comparison results suggested that all these machine learning baselines had worse performance than CAMP. We speculated that the poor performances of these machine learning baselines were mainly because it was relatively hard to predict peptide-protein interactions with only amino acid information, and the latent information under the amino acid sequences was difficult to capture using shallow machine learning methods.

For the binary interaction prediction results, we noticed that CAMP generated relatively variant prediction results under certain cluster settings (Fig. 2). We speculated that this result could be caused by the characteristics of the proteins or the proportion

of unknown or non-standard residues in the sequences. To further investigate these factors, we conducted additional analyses using a five-fold cross-validation procedure on the binary prediction task. In particular, we chose to focus on the "random-split setting" and two specific cases with the largest prediction errors, i.e., the "novel protein setting" with clustering threshold 0.3 and the "novel peptide setting" with clustering threshold 0.4. As shown in Supplementary Fig. 1, we observed that the model performance was not affected much by the proportion of unknown or non-standard amino acids (denoted as "X") in the peptide sequences, with Spearman correlation coefficients -0.16, 0.16 and 0.17 for the "random-split setting", "novel protein setting" and "novel peptide setting", respectively. To further explore the potential biological factors affecting the model performance, we also examined the distributions of AUC scores across the organisms of proteins (Supplementary Fig. 1b), protein families (Supplementary Fig. 1c) and protein domains (Supplementary Fig. 1d). The analysis results indicated that the large prediction errors may result from certain protein families, domains and organisms (e.g., histone and GPCR for protein families, trypsin and kringle for domains and *bovine* for the protein organisms).

Supplementary Table 1. Performance comparison of different machine learning baseline methods. The mean and standard deviation of each method on the benchmark dataset over five folds are shown.

Method	AUC	AUPR
Random forest	0.6289 ± 0.0028	0.3554 ± 0.0080
GBDT	0.5926 ± 0.0019	0.3233 ± 0.0038
XGBoost	0.6261 ± 0.0089	0.3408 ± 0.0158
Logistic regression	0.5589 ± 0.0034	0.2976 ± 0.0047
CAMP	0.8715 ± 0.0052	0.6414 ± 0.0059

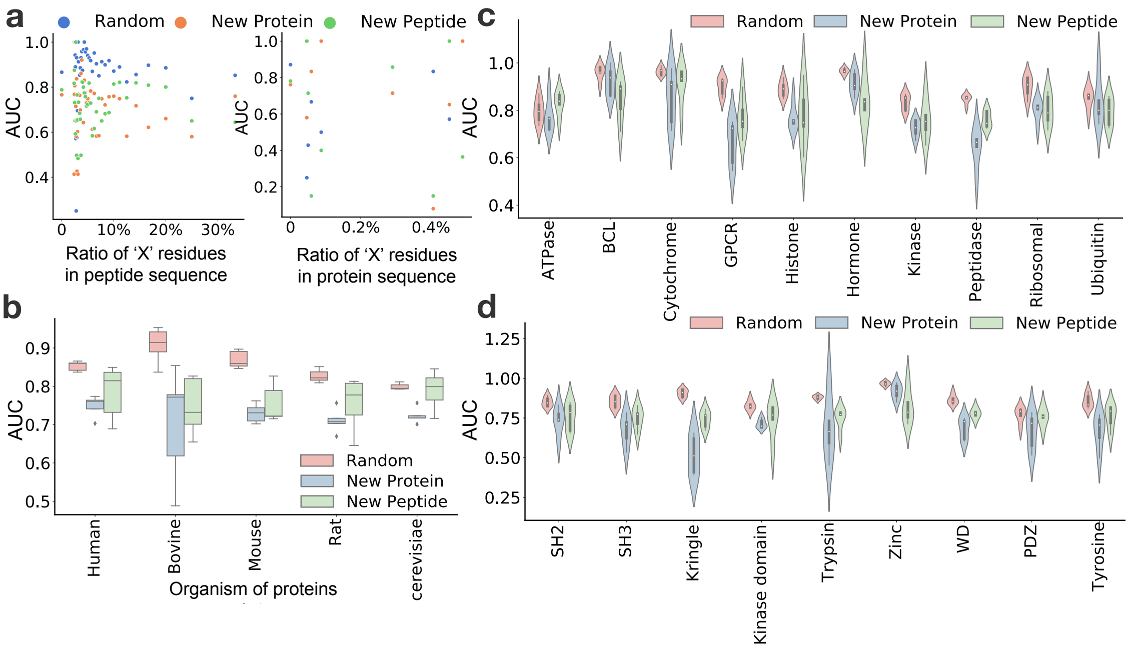

Supplementary Figure 1. Performance of CAMP on the binary prediction task across different factors, related to Fig. 2. a The distribution of AUC scores with different proportions of residues encoded as 'X' in the peptide (protein) sequences. Each data point represents a prediction result on the test dataset using five-fold cross-validation under a specific setting. **b** The distribution of AUC scores in terms of the organisms of proteins. The box plots show the median (middle line), 25th, 75th percentile (box) and the whiskers (extending 1.5 times the interquartile range) as well as outliers (single points). We only showed top five organisms with the highest frequencies in the benchmark dataset, which had 2,901, 497, 365, 194 and 165 interacting pairs for *human*, *mouse*, *cerevisiae*, *bovine* and *rat*, respectively. **c-d** The distributions of AUC scores in terms of protein families and domains, respectively. The violin plots show the median (white dot), the 25th, 75th percentile (thick gray bar in the center) and the thin gray line (extending 1.5 times the interquartile range). We only showed the most common ones in the benchmark dataset, which had 527, 306, 217, 110, 89, 85, 75, 63, 61 and 46 interacting pairs for peptidase, kinase, hormone, histone, ribosomal, ATPase, cytpchrome, ubiquitin, GPCR and serpin, respectively, and 247, 214, 180, 151, 148, 145, 136, 126 and 118 interacting pairs for trypsin, Zinc, kinase domain, SH3, SH2, Kringle, PDZ, WD and tyrosine domains, respectively.

Supplementary Note 2: Ablation studies of the feature encoding scheme and the network architecture for peptide-protein interaction prediction

Here, we demonstrated the importance of the encoded features, the self-attention modules and the binding residue prediction module by conducting a series of ablation studies. We investigated the contributions of these components by comparing the performances of CAMP on the binary interaction prediction with six alternative versions, including removing secondary structure features, physicochemical property features, evolutionary information of the proteins, intrinsic disorder tendencies, the self-attention modules and the peptide binding residue prediction module, respectively.

Here we carried out a five-fold cross-validation procedure on our benchmark dataset and conducted two-tailed student t-tests on AUC and AUPR of each fold to quantify the difference. Supplementary Table 2 showed that the secondary structure features greatly boosted the performance, and adding the evolutionary profiles of proteins as well as the intrinsic disorder scores also brought a significant improvement in pairwise interaction prediction. Although it seemed that introducing physicochemical properties of residues did not bring much increase in AUC, we observed a significant increase in AUPR. For the CAMP model without self-attention modules, both AUC and AUPR were significantly decreased. We also tested the performance of a single-objective version of CAMP, in which the peptide binding residue prediction module was removed. We observed a mild decrease in the performance (i.e., 1.8% and 2.8% in terms of AUC and AUPR, respectively). This difference indicated that adding extra supervise information from peptide binding residue labels can improve CAMP prediction. Furthermore, the predicted binding residues can

also provide useful hints to enhance our understanding of peptide binding mechanisms.

We further examined the performance of CAMP with other attention settings by applying self-attention on different groups of features. As shown in Supplementary Fig. 2, our original attention setting of CAMP achieved better accuracy comparing to the models that applied attention on other features. More specifically, applying self-attention on the amino-acid features achieved better performance over those settings with attention on the categorical features, numeric features and concatenated features (i.e., for each residue, concatenating its both categorical and numeric features) by 7.8%, 7.0% and 8.3% in AUC scores and 17.7%, 19.2% and 21.7% in AUPR scores, respectively. One possible reason was that the amino-acid features may have already contained sufficient information for determining the contributions of individual residues. On the other hand, although the other features can also provide useful information for learning the biological properties of the sequence to infer the binding activities, they were somehow unnecessary for capturing the contributions of individual residue features. The above results demonstrated that applying attention on the amino-acid features was more suitable for our prediction task and can improve the performance to a much greater extent.

	AUC	AUPR	P-values of (AUC, AUPR)
CAMP	0.8715 ± 0.0052	0.6414 ± 0.0059	NA
-no secondary structures	0.8597 ± 0.0018	0.6160 ± 0.0043	(0.00266, 0.00011)
-no physicochemical properties	0.8604 ± 0.0065	0.6263 ± 0.0104	(0.02850, 0.03549)
-no evolutionary matrices	0.8591 ± 0.0052	0.6104 ± 0.0170	(0.00975, 0.02055)
-no intrinsic disorder tendencies	0.8430 ± 0.0064	0.5864 ± 0.0130	(0.00012, 0.00006)
-no attention	0.8412 ± 0.0227	0.5810 ± 0.0404	(0.03150, 0.01818)
-no binding residue prediction	0.8538 ± 0.004	0.6131 ± 0.0127	(0.00069, 0.00373)

Supplementary Table 2. Performance comparison of CAMP with six alternative versions through five-fold cross-validation in a "random-split setting", in which we randomly split the benchmark dataset and used 80% of the dataset to train the model and the remaining 20% to evaluate the performance. We applied the same parameter tuning strategy as described in Supplementary Note 12. The mean and standard deviation of each method on the benchmark dataset over five folds are shown. Also, p-values (two-tailed student t-tests of performance comparison) are reported.

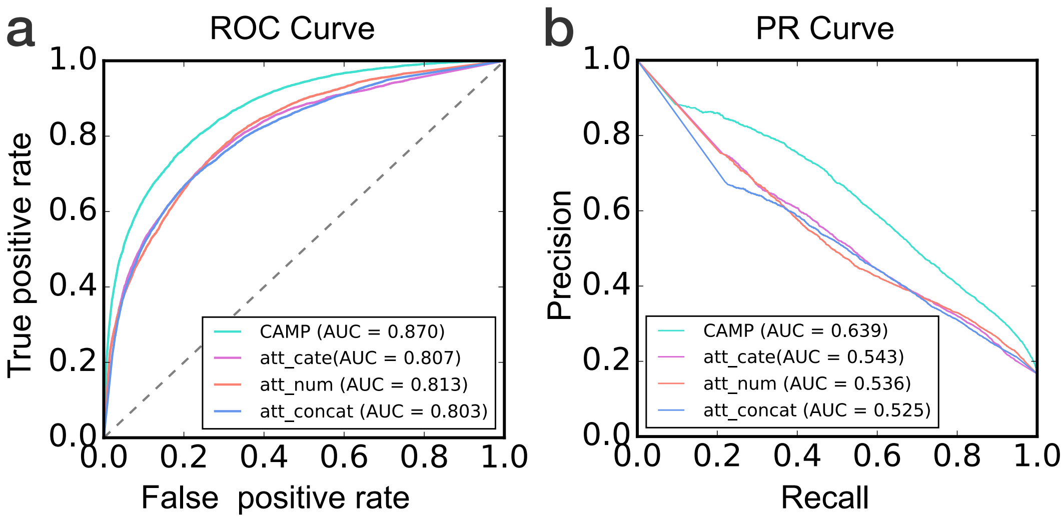

Supplementary Figure 2. Performance evaluation of CAMP with different attention settings on the binary interaction prediction task. a Receiver-operating characteristic (ROC) curves and corresponding area under receiver-operating characteristic (AUC) scores of CAMP with different attention settings. **b** Precision recall (PR) curves and the corresponding area under precision recall (AUPR) scores of CAMP with different attention settings. We conducted five-fold cross-validation under the "random-split setting" on the benchmark dataset. Here, "att-cate" represents the setting of applying the self-attention scheme on the concatenated categorical features, including the amino acid features, the secondary structure features and the physicochemical features; "att-num" represents the setting of applying the self-attention scheme on the concatenated numeric features, including the PSSM features and the intrinsic disorder features; and "att-concat" represents the setting of applying the self-attention scheme on the concatenated categorical and numeric features.

Supplementary Note 3: Additional results on "New insights by characterizing binding residues on peptides"

Here, we examined the performance of peptide binding residue prediction under other three settings, i.e., "novel protein setting", "novel peptide setting" and "novel pair setting". We observed that the performance of peptide binding residue prediction had a decreasing trend as the clustering threshold increased (Supplementary Fig. 3), which was similar to that of the binary interaction prediction task as shown in Fig. 2. As expected, we observed that the performance under the "novel protein setting" largely outperformed the other settings, since the task of predicting peptide binding residues mainly focused on the feature information of peptides, and should be relatively less affected by the protein profiles. This result suggested that CAMP depended more on the peptide features to capture the binding residues involved in the interactions.

To further investigate the impact of partner binding proteins on the peptide binding residue prediction, we examined the predicted binding residues of the same peptide when binding to different partner proteins. Among all the interacting pairs with known peptide binding residues, 942 peptides bind to more than one protein and 332 peptides have different binding residues. Supplementary Fig. 4a shows the distribution of the numbers of distinct binding proteins for individual peptides. The prediction results of the top ten peptides that had the largest numbers of distinct partner proteins showed that CAMP achieved high performance with relatively small variance in these cases (Supplementary Fig. 4b). More specifically, Supplementary Fig. 4c-4e shows three representative cases with the largest numbers of distinct binding proteins in the benchmark dataset. The variance of the true binding residues indicated that these peptides actually have distinct

binding residues when binding to different partner proteins. We observed that the true binding "motifs" and our predicted results had roughly similar distributions (Supplementary Fig. 4c-4e), which further illustrated that CAMP can predict such distinct binding residues for the same peptide when different protein features are fed into our model. We also presented two instances of concrete complex structures (Supplementary Fig. 5) to show that different binding poses can lead to variant binding residues. Overall, the above results demonstrated that CAMP can successfully capture difference between the intrinsic features of distinct proteins binding to the same peptide, which provided another evidence to support the predictive power of CAMP.

In addition, we performed new tests to investigate the contributions of protein information on the peptide binding residue prediction task by removing the input protein features from CAMP. More specifically, for each input peptide, the modified CAMP model first extracted the features through embedding layers, and then exploited three sequential convolutional layers to learn the hidden feature information of the peptide. Finally, it generated an output vector with the same sequence length of the input peptide, where each element implied whether the corresponding position was a binding residue. After removing duplicates, we obtained 5,269 peptide sequences with available binding residue labels. Through a five-fold cross-validation procedure under the "random-split setting", the model achieved an average AUC of 0.6821. On the other hand, when introducing the protein features and adding the supervised labels of binary interactions, the performance of peptide binding residue prediction was significantly improved to 0.806. These results suggested that CAMP can barely identified the correct binding residues when using only peptide features as input. We also illustrated that CAMP was able to yield relatively robust performance with respect to the peptide length and the numbers of binding residues on the peptide (Supplementary Fig. 6).

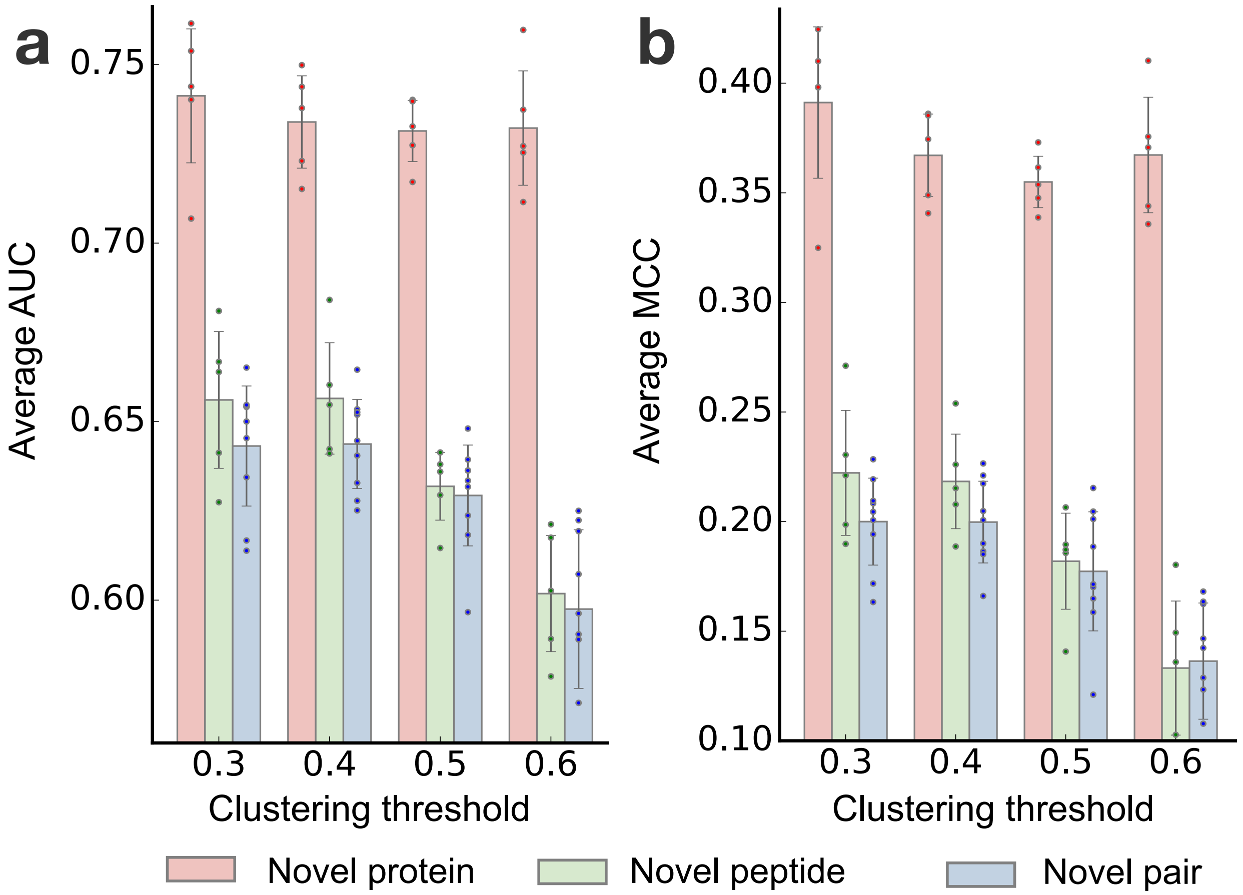

Supplementary Figure 3. The performance of CAMP on peptide binding residue prediction under three settings. For "novel protein setting", $n = 5$, where each data point represents the performance on the corresponding fold under five-fold cross-validation. For "novel peptide setting", $n = 5$, where each data point represents the performance on the corresponding fold under five-fold cross-validation. For "novel pair setting", $n = 9$, where each data point represents the performance on the corresponding data fold under nine-fold cross-validation. Error bars represent mean ± standard deviation. **a** The average AUC scores of CAMP on peptide binding residue prediction through cross-validation under three different settings. **b** The average MCC scores of CAMP on peptide binding residue prediction through cross-validation under three different settings. We adopted the same cross-validation strategy as conducted in our binary interaction prediction task.

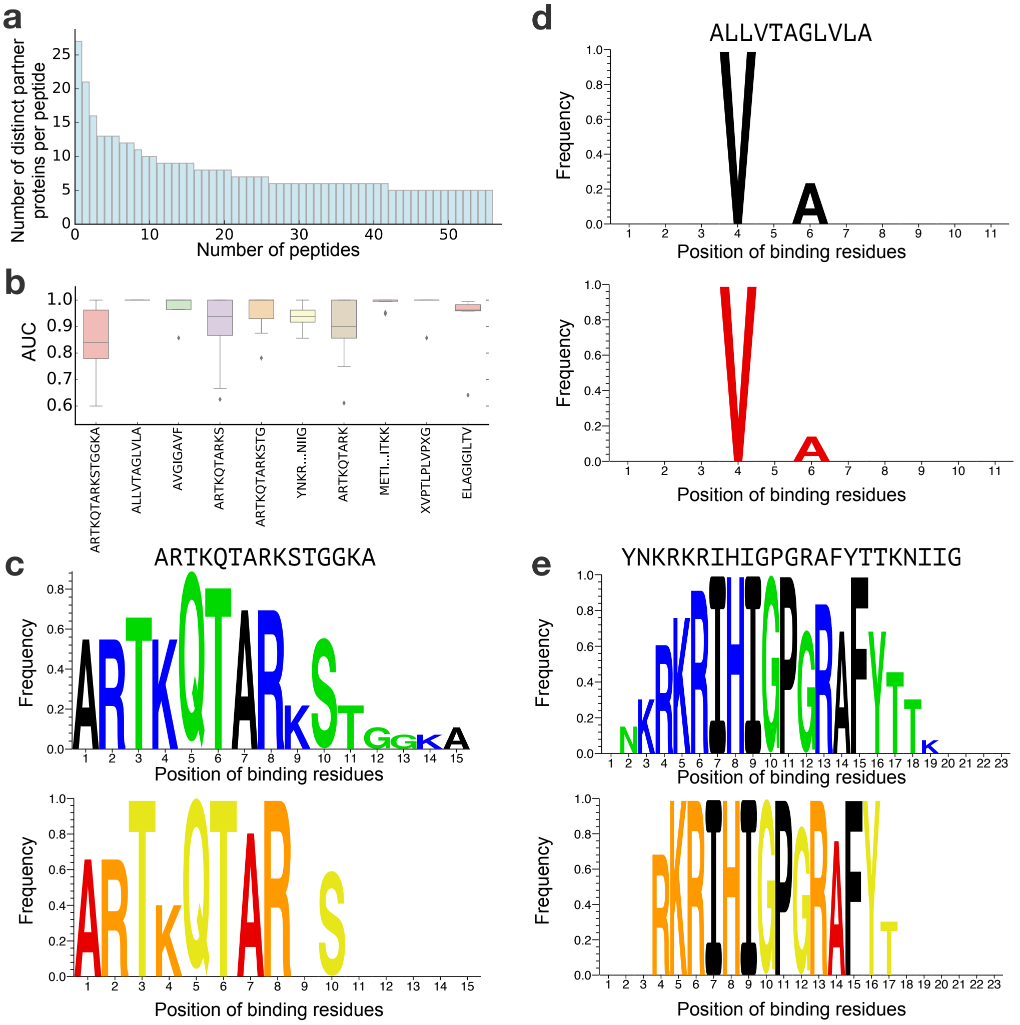

Supplementary Figure 4. The performance of peptide binding residue prediction for peptides binding to different partner proteins through five-fold cross-validation. a The distribution of the numbers of partner proteins for individual peptides. **b** The top 10 peptides with the best performance (in terms of the average AUC score) on predicting binding residues with different binding proteins. $n = 27, 21, 14, 13, 13, 13, 12, 12, 11, 10$ represent the numbers of different binding partner proteins for individual peptides from left to right plotted in the figure. The box plots show the median (middle line), 25th and 75th percentile (the gray box) and the whiskers (extending 1.5 times the interquartile range) as well as the outliers (single dots). **c-e** Three examples of peptide binding residue prediction. The top panel (warm color) shows the true frequency of individual residues along the peptide binding to the partner proteins and the bottom panel (cold color) illustrates the corresponding prediction result.

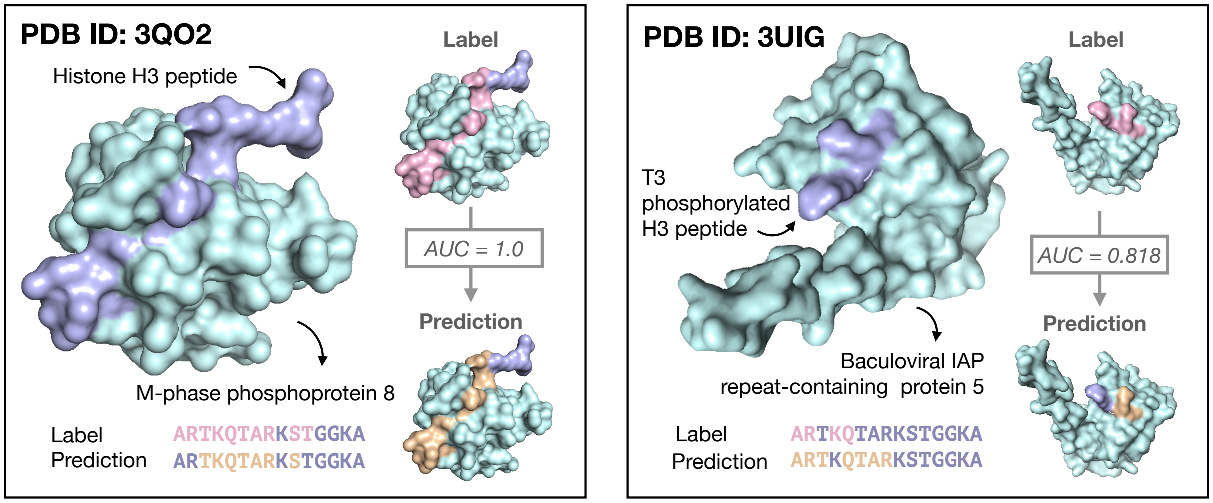

Supplementary Figure 5. Two examples of peptide binding residue predictions by CAMP for the same peptide when binding to different proteins. The PDB complexes were retrieved from the RCSB PDB [1, 2]. The images were generated by PyMOL [3]. The left panel shows the complex structure of MPP8 chromodomain interacting with histone H3 lysine 9 (PDB ID: 3QO2 [`http://doi.org/10.2210/pdb3QO2/pdb`]), and the right panel shows the complex structure of human survivin and T3 phosphorylated H3(1-15) peptide (PDB ID: 3UIG [`http://doi.org/10.2210/pdb3UIG/pdb`]). The protein chains in both complexes are colored in light blue while the peptide chains are colored in light purple. To display the detailed predicted binding sites, we colored the true binding residues pink and the predicted ones wheat, respectively.

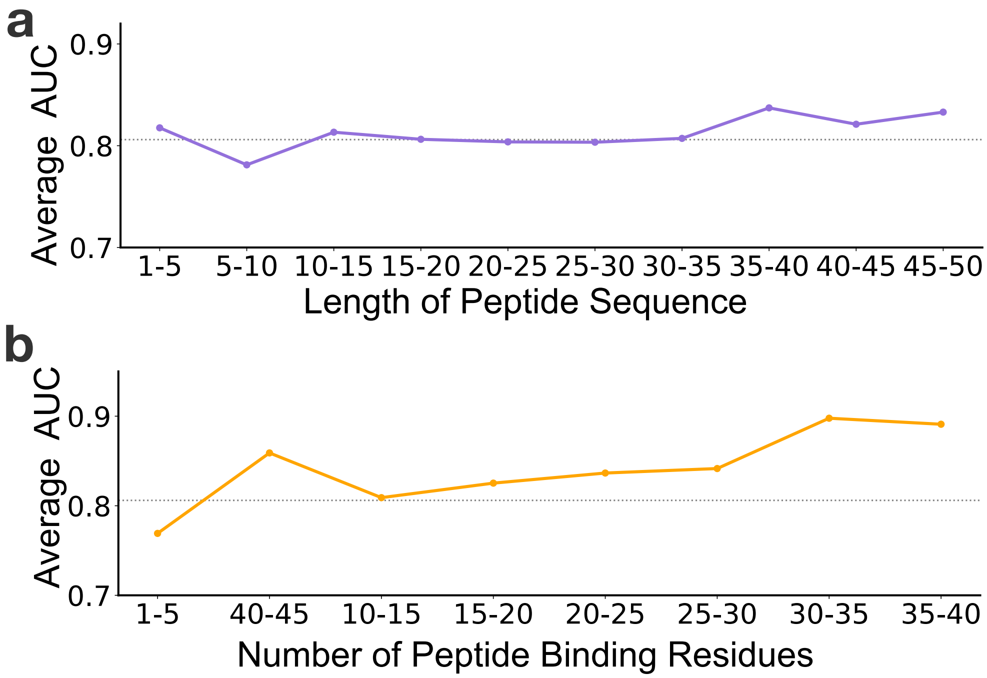

Supplementary Figure 6. The average AUC across various sequence lengths and numbers of peptide binding residues in the sequence. **a** shows that CAMP yields a stable performance over different lengths of peptide sequences and a slight increase of prediction scores for peptides with 35-40 amino acids, which was likely caused by a relatively larger number of samples within this length range. **b** shows that while the number of peptide binding residues increased, the average AUC displayed a slight uptrend, probably due to the extra information provided by the additional interaction information.

Supplementary Note 4: Additional results on "Identifying GLP-1 receptor as a target of Semaglutide and its analogs"

PDB	Peptide Sequence	Prediction Score	Rank of GLP-1R
3IOL	HAEGTFTSDVSSYLEGQAAKEFIAWLVKGRG	0.388	14.47%
4ZGM	HAEGTFTSDVSSYLEGQAAKEFIAWLVRGRG(∗)	0.577	9.97%
5OTT	HXEGXFTSDLSKQMEEEAVRLFIEWLKNGGPSSGAPPPS	0.891	1.32%
5OTU	HXEGXFTSDVSSYLEGQAAKEFIAWLVKGRG	0.770	3.03%
5OTV	HCEGXFTSDVSSYLEGQAAKEFIAWLVKGRG	0.829	3.18%
5OTW	HXEGCFTSDVSSYLEGQAAKEFIAWLVKGRG	0.778	5.38%
5OTX	HCEGCFTSDVSSYLEGQAAKEFIAWLVKGRG	0.729	7.97%

Supplementary Table 3. The prediction scores and ranks of GLP-1R when predicting the protein targets of Semaglutide and its analogs. ∗ denotes Semaglutide.

We further checked the correlation between the numbers of binding residues in the peptides and the corresponding predicted scores (Supplementary Table 3). The Pearson correlation coefficient was 0.4709, which indicated that the predicted scores displayed a moderate correlation with the numbers of binding residues in the peptides. Also, although the first peptide had only one substituted residue, we observed obvious difference of the attention scores between the 13th and 25th positions (Supplementary Fig. 7a). To further investigate the possible reasons that caused such differences, we also compared the input features of individual residues between the two peptides. Although the secondary structures and biophysical properties were not changed much when the lysine was substituted by arginine, the intrinsic disorder features were significantly altered at certain positions, i.e., the long disorder scores (which consider the long stretches of disorder, such

as flexible linkers and loops covering the query residue), the short disorder scores (which consider the short stretches of disorder covering the query residue) and the ANCHOR scores (which indicate the structure flexibility of the query residue). As shown in Supplementary Fig. 7b, the peptides in PDB complexes 3IOL and 4ZGM displayed significant difference in terms of the ANCHOR scores (i.e., structural flexibility), which can well explain the corresponding difference of the attention scores between them. We found that the ANCHOR score of the first peptide in Supplementary Table 3 was generally smaller than that of Semaglutide, which may be the main reason that its prediction score was ranked 5% behind. These results revealed that CAMP was sensitive enough to capture the subtle difference of the peptide features in small modification or substitution along the sequence that may cause variation in structural flexibility and binding activities.

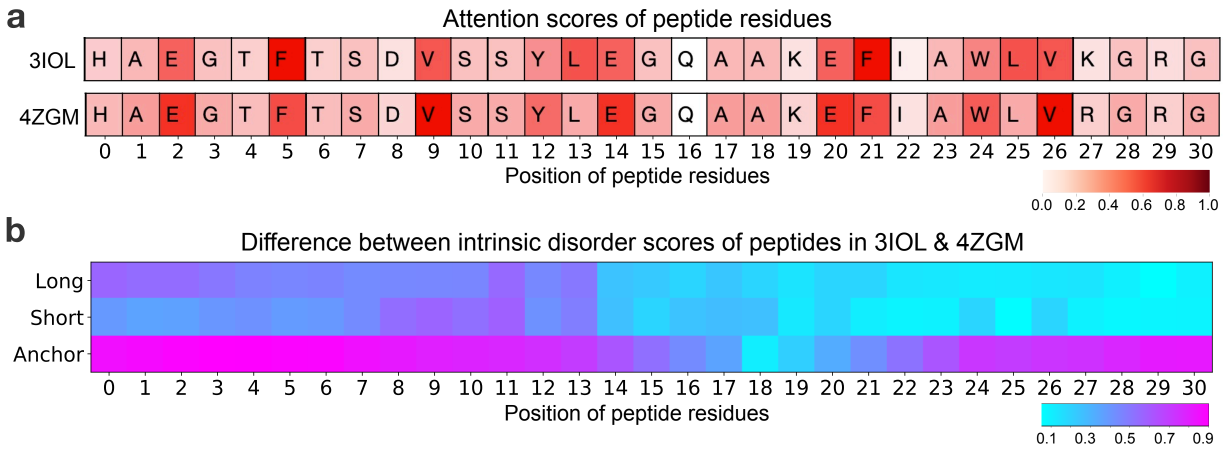

Supplementary Figure 7. The differences of attention scores and intrinsic disorder features between the two peptides interacting with the GLP1 receptor. a The attention scores of individual positions derived from CAMP for the peptides interacting with the GLP1 receptor. The darker the color is, the higher the attention value at the corresponding position is. Although the overall distributions of the attention scores of the two peptides were similar, the attention scores at certain positions (i.e., the 13th and 25th positions) displayed significantly different patterns. **b** The difference of the intrinsic disorder features between the peptides in the two PDB complexes (4ZGM and 3IOL). For each residue, we calculated the differences between the long intrinsic disorder scores (denoted as "Long"), the short intrinsic disorder scores (denoted as "Short") and the ANCHOR scores (denoted as "Anchor"), respectively.

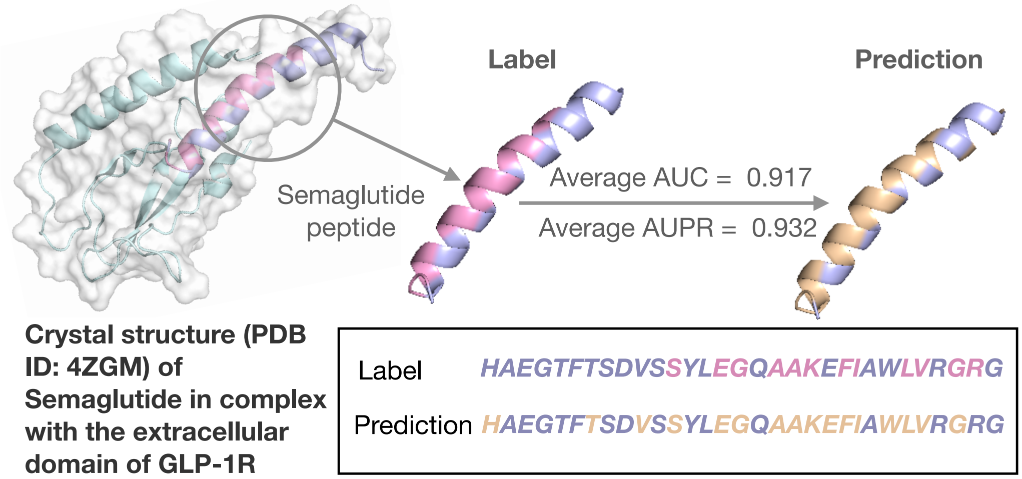

Supplementary Figure 8. The prediction of binding residues of Semaglutide with GLP-1R. The structure of the Semaglutide-GlP-1R complex (PDB ID: 4ZGM [`http://doi.org/10.2210/pdb4ZGM/pdb`]) was retrieved from the RCSB Protein Data Bank (PDB) [1, 2] and the image was generated by PyMOL [3]. The GLP-1 receptor (UniProt ID: P43220) is colored in light blue while Semaglutide is colored in light purple and pink. The Semaglutide peptide binds to its partner protein through 12 residues, which are colored in pink and the binding residues predicted by CAMP are colored in wheat in both sequence and structure visualization. CAMP identified 11/12 of the binding residues with five false positives.

Supplementary Note 5: Additional results on "Generalizability on additional benchmark datasets"

Supplementary Table 4. The statistical information of the additional benchmark datasets. To evaluate the performance of binary interaction prediction task, we also generated the negative samples using the same strategy as described in the Methods section (i.e., shuffling the non-interacting pairs). All these datasets were essentially derived from the Protein Data Bank (PDB). "Num" is the abbreviation of "Number".

Dataset	Num of PDBs	Num of Proteins	Num of Peptides	Model
PPDbench	133	111	110	[4]
LEADS-PEP	53	34	32	[5]
PepSet	185	173	164	[6]
TS251	251	230	222	[7,8]
TS125	125	100	93	[7,9,10]

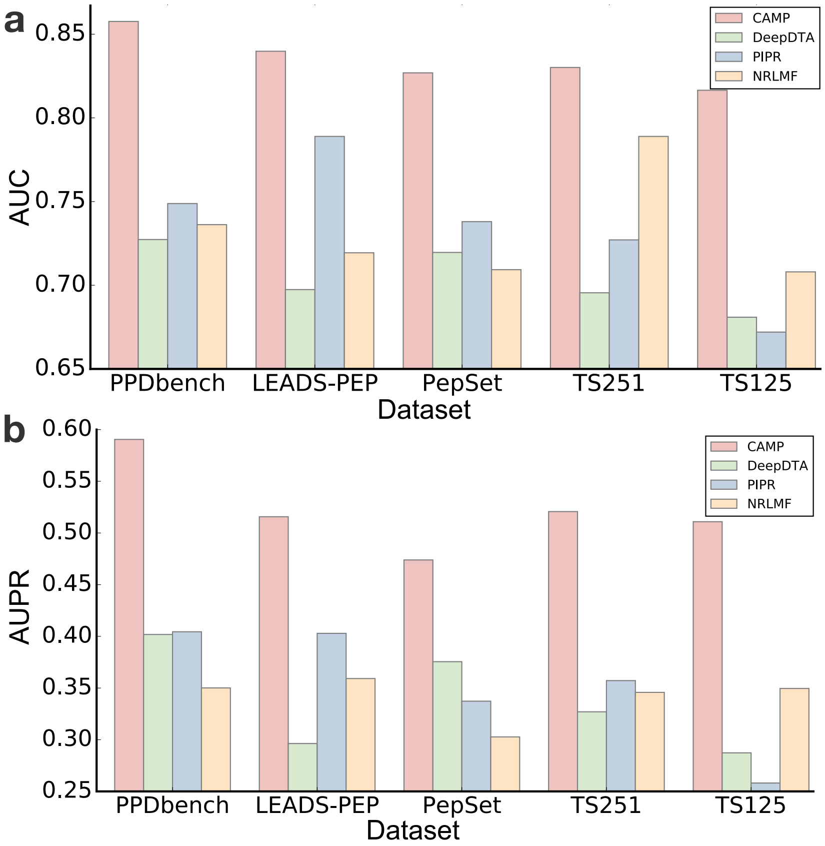

Supplementary Figure 9. Performance evaluation of CAMP and baseline methods on several additional benchmark datasets through five-fold cross-validation. We generated the negative samples using the same strategy as described in the Methods section (i.e., shuffling the non-interacting pairs). **a** AUC scores of CAMP and baseline models. **b** AUPR scores of CAMP and baseline models.

Supplementary Note 6: Additional results on "Extended applications in three related tasks"

For peptide-PBD interaction prediction, we downloaded the data used in HSM (https://github.com/aqlaboratory/hsm), which contained 1,894,338 peptide-PBD pairs in total, covering 482 binding domains and 27,897 peptides with a positive-negative ratio of 1:37.2. In each PBD-peptide pair, the PBD represented a protein binding domain and the peptide represented a short linear sequence in the partner protein where the globular domain is bound to. Next, we conducted the same feature generation procedures as used in our original CAMP model to obtain a group of sequence-based features for both peptides and PBDs. After that, we evaluated the performance of CAMP under exactly the same eight-fold cross-validation setting as used in the original HSM paper [11].The test results shown in Fig. 5 suggested that CAMP can accurately predict PBD-peptide interactions and significantly outperformed the other methods. Therefore, our test results indicated that CAMP can also be successfully extended to predict the interactions between protein domains and linear peptidic sites.

Next, we compared the performance of CAMP with other baselines on the binding affinity prediction task on an affinity dataset derived from PDBbind v2019 [12,13], which contains a high-quality set of protein-ligand complex structures with available binding affinities originated from the RCSB PDB [1,2].We mapped the PDB IDs in this dataset to our benchmark dataset and finally obtained 1,797 peptide-protein pairs with the logarithm of affinity values as labels (data with affinities three standard deviations away from the means were considered outliers and thus removed). The corresponding PDB IDs that we used can be found in Supplementary Table 17 in Supplementary Data. The comparison

results between CAMP and different baselines are shown in Supplementary Table 5.

We also investigated if CAMP can be used for virtual "alanine scanning". For the complex structure of the AF9 YEATS domain binding to the histone H3K9ac peptide (PDB ID: 4TMP [http://doi.org/10.2210/pdb4TMP/pdb]), Li et al. [14] substituted F28, S58, H56, F59, G77, Y78 and D103 around its H3K9ac binding surface with alanine and R8 of H3(1-15)K9ac peptide, respectively. For another complex structure of the ZMYND11 Bromo-PWWP domain binding to histone H3.1K36me3 (PDB: 4N4H [http://doi.org/10.2210/pdb4N4H/pdb]), Wen et al. [15] substituted R168, D307, E251, N266, W294 and F310 of the ZMYND11 Bromo-PWWP domain with alanine individually. In both studies, the authors conducted quantitative isothermal titration calorimetry (ITC) analyses to assess the binding affinities of each mutation. Here, we further trained CAMP on the aforementioned dataset derived from PDBbind v2019 [12] and the binding affinity data between wild-type proteins and peptides derived from [14, 15]. Then we used the trained model to predict the affinities for the mutant sequences. The results of these two case studies are shown in Supplementary Fig. 10.

Supplementary Table 5. Performance comparison of different methods on the binding affinity prediction task. We applied the same parameter tuning strategy as described in Supplementary Note 12. The means of individual methods on the affinity dataset over five folds are shown. Note that the Root Mean Square Errors (RMSEs) and Pearson correlation coefficients were both calculated based on the logarithms of the experimentally-measured affinities and the prediction scores.

Method	RMSE	Pearson Correlation
CAMP	1.1096	0.6732
DeepDTA	1.1676	0.6338
Random Forest	1.2606	0.5188
AutoDock CrankPep	13.9314	-0.0506

For virtual screening of peptides, following the previous framework of molecular docking for small molecule virtual screening [16], we constructed a docking test set to investi-

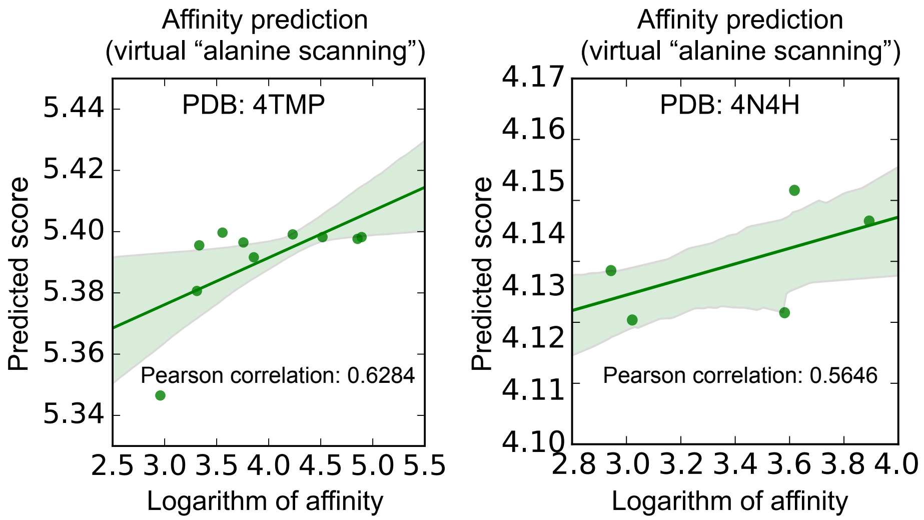

Supplementary Figure 10. Performance of CAMP on two virtual "alanine scanning" experiments. The Pearson correlation coefficients between the logarithms of binding affinities and predicted scores for the PDB complex 4TMP and 4N4H were 0.6284 and 0.5646, respectively. In each plot, a linear regression model (centred green line) is fitted on the data points and the translucent band around the regression line denotes the 80% confidence interval for the regression.

gate the performance of CAMP and several peptide docking methods. We first obtained the active data by adopting the 3D peptide-protein complexes from LEADS-PEP [5], a benchmark used for the assessment of peptide docking performance. There are 53 peptide-protein complexes in LEADS-PEP, covering 34 distinct proteins and 32 different peptides. Then we generated the decoys by randomly sampling from all the shuffled pairs of non-binding proteins and peptides. We evaluated CAMP using five-fold cross-validation and reported the test performance on the active and decoy data. For the docking methods, we simply ran the corresponding docking programs on the active and decoy data, and reported the AUC scores between the docking scores and the binary labels (1 for active and 0 for decoy).

Our comparison results showed that CAMP significantly outperformed the peptide docking based methods (Supplementary Table 6). We also observed that CABS-Dock [17]

and MDockPeP [18] failed to distinguish between the active and decoy pairs. We speculated that the reason might be that these two methods only require peptide sequences and protein structures as input, thus lacking crucial information of the true ligand structures that are necessary for docking. Since AutoDock CrankPep v1.0 [19] takes both solved crystal structures of the protein and peptide as input, it had a slightly better performance than CABS-Dock and MDockPeP. On the other hand, GalaxyPepDock [20] yielded better prediction results than the other docking baselines, which was probably due to the fact that GalaxyPepDock need to select templates based on the structures and interaction similarity from the PepBind database [21], and there was overlap between PepBind and LEADS-PEP datasets since they were both derived from the RCSB PDB [1, 2]. Such overlap was inevitable when we ran GalaxyPepDock through the web server, which thus may lead to an over-optimistic performance. All the above results demonstrated that CAMP can provide a better tool for virtual screening of peptides.

Supplementary Table 6. Performance evaluation of CAMP and the structure-based docking methods (in which the scoring functions are also described) on the LEADS-PEP test set.

Method	AUC	Scoring Function Type
CAMP	0.8398	Deep learning based scoring function
AutoDock CrankPep [19]	0.5713	Empirical scoring function
GalaxyPepDock [20]	0.7728	Energy-based scoring function
CABS-Dock [17]	0.4580	Clustering-based scoring function
MDockPeP [18]	0.4931	Statistical potential-based scoring function

Supplementary Note 7: Construction of sequence-based feature profiles

Residue-level structural and physicochemical properties

For amino acid representation, each residue is denoted by a letter from a 21-letter alphabet $\mathcal{A}$, containing a vocabulary of 21 types of amino acids (i.e., 20 canonical amino acids and a letter 'X' for any unknown or non-standard amino acid).

Here, we used SSPro [22] to predict a 3-class secondary structure type (Supplementary Table 7) for each residue, which is denoted by a letter chosen from a three-letter alphabet to represent the class of the predicted secondary structure (i.e., helix, strand and the rest). Next, we define an alphabet of 63 elements to describe the combination of the predicted secondary structure type and the amino acid type of each residue. Each combination is encoded with an integer between 1 and 63.

The physicochemical property representation is to encode the physicochemical features of the R group of each residue, denoted by a letter from a seven-letter alphabet based on the combination of polarity and the hydropathy index (a metric measuring the hydrophilicity and hydrophobicity of the R groups). This index reflects the free energy of the transfer of an amino acid side chain from a hydrophobic solvent to water [23]. The positive value indicates that the transfer is unfavorable for amino acids with nonpolar side chains and the negative value indicates that the transfer is favorable for charged or polar amino acid side chains. We therefore define an alphabet of seven elements to describe the combination of polarity and the hydropathy index of each amino acid side chain (Supplementary Table 8). In particular, each combination is encoded with an integer between 1 and 7.

Protein evolutionary information

Given a protein sequence, we use its normalized PSSM matrix derived by PSI-blast [24,25] ($iteration = 3$ and $E - value = 0.001$). Here the parameters (the number of iterations and the threshold of E-value) have been proved to work well for generating effective feature profiles from the protein sequences [25] (More details about the determination of hyper-parameters can be found in Supplementary Note 12.).

Intrinsic disorder tendencies to form contacts

The intrinsic disorder scores represent the tendencies of disordered amino acid pairs to form contacts, ranging from 0 (complete order) to 1 (complete disorder). In particular, we adopt two kinds of intrinsic disorder scores, i.e., the long disorder score (considering the long disordered regions such as disordered domains) and the short disorder score (considering the short stretches of disorder such as flexible linkers and loops) [26]. We also consider the ANCHOR score (a normalized score between 0 and 1), which represents the probability of a given residue to be part of a disorder binding region, thus indicating the structure flexibility of the query residue and its neighbors [27].

Amino acid	One-letter representation
- Standard abbreviation	Gly:G, Ala:A, ...,Glu:E
- Unknown/non-standard	X
Secondary structure	One-letter representation
- Strand	E
- Helix	H
- Rest	C

Supplementary Table 7. Representations of individual amino acid types and the secondary structure elements

Polarity	Positive/negative of Hydropathy Index	Amino-acid members	Number of members
Nonpolar	Positive	Ala, Phe, Ile, Met, Leu, Pro, Val	7
Nonpolar	Negative	Gly, Trp	2
Polar-uncharged	Positive	Cys	1
Polar-uncharged	Negative	Asn, Gln, Ser, Thr, Tyr	5
Negatively-charged	Negative	Asp, Glu	2
Positively-charged	Negative	Lys, His, Arg	3
Unknown	Unknown	Otherwise	NA

Supplementary Table 8. Physicochemical property representations of individual residues in protein or peptide sequences are denoted by the combination of polarity and Hydropathy Index of the R Groups.

Supplementary Note 8: Cluster-based cross-validation

In the real peptide-protein interaction prediction setting, the data redundancy problem caused by similar proteins or peptides may lead to "easy predictions", which could mislead the performance evaluation of different algorithms. To conduct an objective evaluation, we followed the same strategy as in MONN [28] and used a cluster-based strategy for cross-validation. The clustering threshold means the minimal distance between any two clusters by a single-linkage clustering algorithm [29]. More specifically, the distance between two peptides (proteins) p_i and p_j is defined as

$$d_{ij} = 1 - \frac{SW(p_i, p_j)}{\sqrt{(SW(p_i, p_i)SW(p_j, p_j))}}, \tag{1}$$

where SW$(\cdot, \cdot)$ stands for the Smith-Waterman alignment score (https://github.com/mengyao/Complete-Striped-Smith-Waterman-Library) between two

sequences.

Our analyses showed that the numbers of clusters and the corresponding sizes of the maximum clusters changed as the clustering threshold increased from 0.1 to 0.9 with step size 0.1 (Supplementary Table 9 and 10). Since the threshold determines the minimal distance between clusters, a higher threshold generally indicates the relatively less similarity of any two proteins (peptides) in different clusters. Thus, we seeked for thresholds that were large enough to distinguish the proteins (peptides) between the training and test sets, but cannot be too large as extremely large clustering thresholds may lead to a small number of clusters and extremely large cluster sizes (Supplementary Table 9 and 10), which may thus disrupt the normal data splitting process during five-fold cross validation. On the other hand, splitting with too small thresholds were not acceptable either, as it can lead to a nearly random splitting result. With the above consideration, we therefore mainly evaluated the results using the clustering thresholds from [0.3, 0.4, 0.5, 0.6].

Threshold	Number of clusters	max cluster size
0.1	2822	24
0.2	2453	169
0.3	2291	252
0.4	2137	565
0.5	1981	566
0.6	1786	566
0.7	1535	714
0.8	169	3182
0.9	1	3412

Supplementary Table 9. The numbers of clusters and the max cluster sizes of proteins in our benchmark dataset under different clustering thresholds. There were in total 3,412 distinct proteins in our benchmark dataset.

For the "novel protein setting" and the "novel peptide setting", we conducted five-fold cross-validation on the sequence clusters instead of directly splitting the sequences

Threshold	Number of clusters	max cluster size
0.1	4270	76
0.2	3544	159
0.3	3085	166
0.4	2486	1209
0.5	1470	3001
0.6	596	4405
0.7	24	5309
0.8	1	5339
0.9	1	5339

Supplementary Table 10. The numbers of clusters and the max cluster sizes of peptides under different clustering thresholds. There were in total 5,339 distinct peptides in our benchmark dataset.

of proteins or peptides. Here, the proportion of validation set was approximately 20%, since the data amount within each cluster was not always evenly distributed. For the "novel pair setting", we conducted cross-validation on both protein and peptide clusters. In particular, we first split protein clusters into three grids, and then within each grid, we further split the peptide clusters into three grids. In this way, we divided the dataset into nine grids for a nine-fold cross-validation procedure. We chose the data of a single grid as a validation set and the remaining four grids that did not have any overlapped protein or peptide cluster as the training set. In such a test setting, there was not any shared protein or peptide cluster across training and test sets.

Supplementary Note 9: Evaluation metrics

To evaluate the performance of CAMP on peptide binding residue prediction, we used the average AUC and average Matthews correlation coefficient (MCC) on our constructed benchmark dataset (Fig. 3a and 3b) and an independent test set (Fig. 4c and 4d). In particular, for the peptide binding residue prediction of a dataset containing N peptide-

protein pairs, the average AUC is defined as

$$\text{Average AUC} = \frac{1}{N}\sum_{i=1}^{N}\text{AUC}(i), \tag{2}$$

where AUC(i) represents the area under the ROC curve of the i-th peptide-protein pair, which is calculated from the true binding vector b_{pep} and the predicted binding vector b'_{pep} of the peptide.

The Matthews correlation coefficient (MCC) is defined as

$$\text{MCC} = \frac{\text{TP} \times \text{TN} - \text{FP} \times \text{FN}}{\sqrt{(\text{TP}+\text{FP})(\text{TP}+\text{FN})(\text{TN}+\text{FP})(\text{TN}+\text{FN})}}, \tag{3}$$

where TP (true positive) is the number of binding residues that are correctly predicted, TN (true negative) is the number of residues that are not involved in binding activities and are correctly predicted, FP (false positive) is the number of residues that are not involved in binding activities but are predicted as binding residues incorrectly, and FN (false negative) is the number of residues that are binding residues but are predicted incorrectly. MCC ranges from -1 to 1 and the value of zero represents a random prediction. Higher values of MCC indicate better prediction.

Then, the average MCC is defined as

$$\text{Average MCC} = \frac{1}{N}\sum_{i=1}^{N}\text{MCC}(i), \tag{4}$$

where MCC(i) represents the Matthews correlation coefficient (MCC) of the i-th peptide-protein pair, which is also calculated from the true binding vector b_{pep} and the predicted binding vector b'_{pep} (defined in the "Methods" section) of the peptide.

Supplementary Note 10: Additional details of datasets

When constructing the benchmark dataset, we excluded a specific class of antigen peptides binding to the major histocompatibility complex (MHC) molecules, since they possess specific binding mechanisms that may not be easily generalized to common peptide-protein interactions. For example, previous structural studies of antigen peptides [30–32] showed that both N and C termini of a peptide play a more important role in peptide-MHC interaction, since these two ends tend to be located in the peptide binding grooves. Systematic studies [33] also revealed that amino acids at certain positions (also known as anchors) in the antigen peptide sequences are highly conservative in binding with MHC molecules. Taken these unique binding characteristics into account, modeling the peptide-MHC interactions is often considered as a separate problem, and so far a number of computational methods have been developed to successfully predict their binding patterns [34–36]. Here, since we mainly aimed at developing a universal framework to predict general peptide-protein interactions, we excluded the binding data between antigen peptides and MHC molecules.

In addition, to maintain the high quality of the constructed dataset, we excluded those pairs that contained peptide sequences with more than 20% unknown or non-standard amino acids, or protein sequences that were longer than 5,000 amino acids as (this threshold could cover more than 99% of the protein sequences). For an input peptide sequence with gapped regions, our framework uses 'X' (i.e., representation of a non-standard or unknown amino acid) to impute the gapped positions for the downstream prediction. In fact, the peptide sequences in our benchmark dataset did not contain any gapped region, probably because this dataset had been carefully manually curated from the RCSB PDB database.

We set the maximum length of peptides to be 50, and those with less than 50 residues are zero-padded. For instance, a peptide sequence containing eight residues is encoded into eight integers from the 1st to 8th positions and the 9th-50th positions are all padded with zeros. During the training process, features in the padded positions are masked to avoid their interference on the calculation of the losses and gradients. We choose the maximum length of proteins to cover at least 80% of the intact proteins in our benchmark dataset based on the computational experiments illustrated in Supplementary Table 11 and the truncating strategy from previous study [37]. Protein sequences longer than the maximum length were truncated and protein sequences shorter than the maximum length were zero-padded.

Truncating Portion (%)	AUC	AUPR
70	0.8580±0.0163	0.6153±0.0249
80	0.8715±0.0052	0.6414±0.0059
90	0.8422±0.0185	0.5874±0.03165
100	0.8296±0.01890	0.5508±0.0392

Supplementary Table 11. The performance of CAMP on binary peptide-protein interaction prediction with different truncating lengths of the protein sequences under five-fold cross-validation. The mean and standard deviation of AUC and AUPR of each test are shown.

RCSB PDB dataset

As shown in Supplementary Fig. 11, for each peptide-protein pair, the peptide sequence was directly obtained from the RCSB PDB [1, 2] with binding residues derived from PepBDB [38] and the protein sequence was obtained by mapping to UniProt [39]. The corresponding PDB IDs that we used for training and testing can be found in Supplementary Table 12 and 13 in Supplementary Data, respectively. The corresponding UniProt IDs that we used for training and testing can be found in Supplementary Table 15 and 16 in Supple-

mentary Data, respectively. We first downloaded all complexes containing peptides as ligands from the RCSB PDB released by September 2019. Then we used the Protein Ligand Interaction Predictor (PLIP) program [40][http://github.com/ssalentin/plip] to extract the interacting chains of peptide and protein sequences from the complex structures. Given a complex structure, PLIP recognizes seven types of non-covalent interactions, including hydrogen bonds, hydrophobic interactions, pi-stackings, pi-cations, salt bridges, water bridges and halogen bonds. A residue from the peptide and another one from the protein, with at least one non-covalent interaction was considered as an interacting pair. We then retrieved the corresponding interacting labels from PepBDB [38], a structure database of peptide-protein complexes derived from the RCSB Protein Data Bank (PDB) [1, 2], which contains the peptide residues involved in hydrogen bonds and and hydrophobic contacts with the partner proteins. The peptide binding residues detected by PepBDB were then mapped to the peptide sequences (which were annotated from the RSCB PDB) using an alignment tool based on the Smith-Waterman algorithm [41] [https://github.com/mengyao/Complete-Striped-Smith-Waterman-Library]. To achieve the high quality of the data, we only kept those peptide sequences with at least 80% matched residues. In total, we collected 7,233 peptide-protein pairs with 3,318 distinct protein sequences and 5,283 distinct peptide sequences, and 90.99% of the pairs had labels of peptide binding residues.

DrugBank dataset

We also collected all drugs belonging to the "peptides category" as well as their target from DrugBank [42–46]. For these PepPIs, peptide sequences were obtained from PubChem [47] and protein sequences were obtained by mapping to UniProt [39]. The corresponding DrugBank IDs and UniProt IDs that we used can be found in Supplementary Table 14

and Table 15 in Supplementary Data. In total, we collected 196 drug-target pairs from DrugBank, including 124 and 61 protein and peptide sequences, respectively.

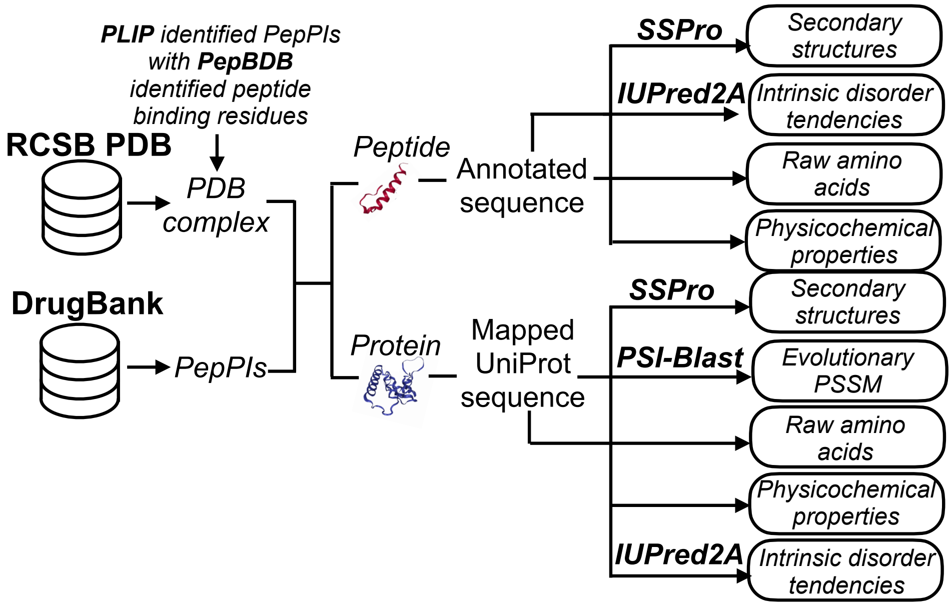

Supplementary Figure 11. The data curation and feature generation procedures. We first obtained all PDB complexes containing peptides as ligands from the RCSB PDB [1, 2] and all peptide drugs with corresponding targets from DrugBank [42–46]. Then for the peptide-protein pairs from the PDB, we used PLIP [40] to identify non-covalent interactions. Any peptide-protein pairs involved such interactions were kept as interacting pairs. we also downloaded the corresponding labels of peptide binding residues from PepBDB [38]. Next, we mapped the protein sequences from the PDB and DrugBank to UniProt [39] to achieve unified inputs of protein sequences and then generated comprehensive feature profiles based on primary sequences of peptides and proteins, including amino acid representations, secondary structures, physicochemical properties, intrinsic disorder tendencies and evolutionary PSSMs.

Supplementary Note 11: The convolution neural network

Each CNN module consists of three layers including the convolution layer, the rectified linear unit (ReLU) layer and the max pooling layer. In our model, the initial feature input of the CNN module of the protein (peptide) is an $N_k \times N_d$ feature array $\mathbf{F}$, where N_k is the length of the input protein (peptide) sequence, and N_d is the dimension of features in each residue position. The convolution layer uses a sliding window of size m along the array F to convert $\mathbf{F}$ to an $[N_k] \times d$ array $\mathbf{H}$, where d is the number of filters. Let $H_{i,k}$ represent the score of the filter k for position i in the array $\mathbf{F}$, and $W_{k,j,l}$ denote the coefficient of filter k at residue position j and feature l. Then the convolution layer computes the function

$$H_{i,k} = ReLU(\sum_{m}^{j=1} \sum_{N_d}^{l=1} W_{k,j,l} F_{i+j,l}), \tag{5}$$

where the column $X_{\cdot,k}$ is a 1-dimensional filter and $ReLU(X) = max(X, 0)$ is the activation function. Finally, the max pooling layer takes the output of the last convolution block to reduce the feature dimension.

Supplementary Note 12: Hyper-parameter selection

PSSM thresholds for feature generation

For constructing the Position-specific scoring matrix (PSSM) profiles of proteins, we ran PSI-BLAST with $iteration = 3$ and $E - value = 0.001$ for multiple alignment, where $iteration$ represents the maximum number of iterations to search for the query protein

through the database and *E-value* represents the significance value of the multiple alignment [24]. Such a default parameter setting of PSI-BLAST had been proved to work well for generating the protein profiles in previous studies, such as [48, 49].

Hyper-parameters of prediction models

There are several hyper-parameters of CAMP, such as learning rate, the number of epochs, the number of filters and the kernel size in the convolution layers, and the size of fully connected layers. Note that due to the huge search space, it would be difficult and time-consuming to find the optimal setting of all these hyper-parameters. Therefore, we only optimize four hyper-parameters, including the number of filters in convolution layers, the size of fully connected layers, the learning rate and the coefficient of the binding residue prediction loss λ through a five-fold cross-validation procedure using a grid search approach. In particular, the search grid for the number of filters in convolution layers is [32,64,128], the grid for the size of fully connected layers is [128,256,512,1024], the grid for the learning rate is [0.0001,0.0005,0.001] and the grid for λ is [0.01,0.1,1,10]. The hyper-parameter setting with the best AUC score over the validation set was selected. The similar searching strategy was also used for other baseline models mentioned in the Results for a fair comparison. In particular, for DeepDTA [37], we conducted a grid search to determine the best combination of hyper-parameters, including the length of sequence window from [4,6,8,12], and we used 100 as the maximum number of epochs, which was the default value from the original paper. For PIPR [50], we conducted the same grid search strategy on the hyper-parameters as in the original paper [50], which chose the dimension of hidden states from [10,25,50] and the number of recurrent convolutional neural network (RCNN) units from [1,2,3,4]. We chose 50 as the default value for the maximum number of epoches. For NRLMF [51], we chose the optimal regulation parameters and the learning

rate from [2^{-5},2^{-4},2^{-3},$\cdots$,2^{2}], [2^{-3},2^{-2},$\cdots$,2^{0}], respectively.

Supplementary References

1. Berman, H. M. et al. The Protein Data Bank. *Nucleic Acids Res.* **28**, 235–242 (2000).

2. Burley, S. K. et al. RCSB Protein Data Bank: biological macromolecular structures enabling research and education in fundamental biology, biomedicine, biotechnology and energy. *Nucleic Acids Res.* **47**, D464–D474 (2019).

3. Schrödinger, LLC The PyMOL Molecular Graphics System, Version 1.8, Schrödinger, LLC. (2015).

4. Agrawal, P. et al. Benchmarking of different molecular docking methods for protein-peptide docking. *BMC Bioinformatics* **19**, 426 (2019)

5. Hauser, A. S. & Windshügel, B. LEADS-PEP: A Benchmark Data Set for Assessment of Peptide Docking Performance. *J Chem Inf Model* **56**, 188-200 (2016)

6. Weng, G. et al. Comprehensive Evaluation of Fourteen Docking Programs on Protein-Peptide Complexes. *J Chem Theory Comput,* **16**, 3959-3969 (2020).

7. Abdin O., Wen H. & Kim PM. PepNN: a deep attention model for the identification of peptide binding sites. Preprint at https://biorxiv.org/content/10.1101/2021.01.10.426132v1 (2021)

8. Johansson-Åkhe, I., Mirabello, C. & Wallner, B. Predicting protein-peptide interaction sites using distant protein complexes as structural templates. *Sci. Rep.* **9**, 4267 (2019).

9. Taherzadeh, G., Zhou, Y., Liew, A. W.-C. & Yang, Y. Structure-based prediction of protein- peptide binding regions using Random Forest. *Bioinformatics* **34**, 477–484 (2018).

10. Wardah, W. et al. Predicting protein-peptide binding sites with a deep convolutional neural network. *J. Theor. Biol.* **496**, 110278 (2020).

11. Cunningham, J. M., Koytiger, G., Sorger, P. K. & AlQuraishi, M. Biophysical prediction of protein–peptide interactions and signaling networks using machine learning. *Nat. Methods* **17**, 175–183 (2020).

12. Wang, R., Fang, X., Lu, Y., Yang, C. Y.& Wang, S. The PDBbind database: methodologies and updates. *J Med Chem* **48**, 4111-4119 (2005)

13. Liu, Z. et al. Forging the Basis for Developing Protein-Ligand Interaction Scoring Functions. *Acc Chem Res* **50**, 302-309 (2017)

14. Li, Y. et al. AF9 YEATS domain links histone acetylation to DOT1L-mediated H3K79 methylation. *Cell* **159**, 558-571 (2014)

15. Wen, H. et al. ZMYND11 links histone H3.3K36me3 to transcription elongation and tumour suppression. *Nature* **508**, 263-268 (2014)

16. Mysinger, M. M., Carchia, M., Irwin, J. J. & Shoichet, B. K. Directory of useful decoys, enhanced (DUD-E): better ligands and decoys for better benchmarking. *J Med Chem* **55**, 6582-6594 (2012)

17. Kurcinski, M., Jamroz, M., Blaszczyk, M., Kolinski, A. & Kmiecik, S. CABS-dock web server for the flexible docking of peptides to proteins without prior knowledge of the binding site. *Nucleic Acids Res* **43**, W419-424 (2015)

18. Xu, X., Yan, C. & Zou, X. MDockPeP: An ab-initio protein-peptide docking server. *J Comput Chem* **39**, 2409-2413 (2018)

19. Zhang, Y. & Sanner, M. F. AutoDock CrankPep: combining folding and docking to predict protein-peptide complexes. *Bioinformatics* **35**, 5121-5127 (2019)

20. Lee, H., Heo, L., Lee, M. S. & Seok, C. GalaxyPepDock: a protein-peptide docking tool based on interaction similarity and energy optimization. *Nucleic Acids Res* **43**, W431-435 (2015)

21. Das, A. A., Sharma, O. P., Kumar, M. S., Krishna, R. & Mathur, P. P. PepBind: A Comprehensive Database and Computational Tool for Analysis of Protein–peptide Interactions. *Genomics Proteomics Bioinformatics* **11**, 241–246 (2013).

22. Magnan, C. N. & Baldi, P. SSpro/ACCpro 5: almost perfect prediction of protein secondary structure and relative solvent accessibility using profiles, machine learning and structural similarity. *Bioinformatics* **30**, 2592–2597 (2014).

23. L., N. D., Lehninger, A. L., Nelson, D. L., Cox, M. M. & University Michael M Cox *Lehninger Principles of Biochemistry.* (W. H. Freeman, 2005).

24. Madeira, F. et al. The EMBL-EBI search and sequence analysis tools APIs in 2019. *Nucleic Acids Res.* **47**, W636–W641 (2019)

25. Hamp, T. & Rost, B. Evolutionary profiles improve protein-protein interaction prediction from sequence. *Bioinformatics* **31**, 1945–1950 (2015).

26. Dosztányi, Z., Csizmok, V., Tompa, P.& Simon, I. IUPred: web server for the prediction of intrinsically unstructured regions of proteins based on estimated energy content. *Bioinformatics* **21**, 3433–3434 (2005).

27. Mészáros, B., Erdos, G. Dosztányi, Z. IUPred2A: context-dependent prediction of protein disorder as a function of redox state and protein binding. *Nucleic Acids Res.* **46**, W329–W337 (2018).

28. Li, S. et al. MONN: A Multi-objective Neural Network for Predicting Compound-Protein Interactions and Affinities. *Cell Systems* **10**, 308–322.e11 (2020).

29. Gower, J. C. & Ross, G. J. S. Minimum spanning trees and single linkage cluster analysis. *J. R. Stat. Soc. Ser. C Appl. Stat.* **18**, 54 (1969).

30. Engelhard, V. H. Structure of Peptides Associated with Class I and Class II MHC Molecules. *Annual Review of Immunology* **12**, 181–207 (1994).

31. Madden, D. R. The three-dimensional structure of peptide-MHC complexes. *Annu Rev Immunol* **13**, 587-622 (1995)

32. Liu, Q. J. & Gao, B. Manipulation of MHC-I/TCR interaction for immune therapy. *Cell Mol Immunol* **5**, 171-182 (2008)

33. Peters, B., Nielsen, M. & Sette, A T Cell Epitope Predictions. *Annu Rev Immunol* **38**, 123-145 (2020)

34. Hu, Y. et al. ACME: pan-specific peptide-MHC class I binding prediction through attention-based deep neural networks. *Bioinformatics* **35**, 4946–4954 (2019).

35. Jurtz, V. et al. NetMHCpan-4.0: Improved Peptide-MHC Class I Interaction Predictions Integrating Eluted Ligand and Peptide Binding Affinity Data. *J Immunol* **199**, 3360-3368 (2017).

36. O'Donnell, T. J., Rubinsteyn, A. & Laserson, U Improved Pan-Allele Prediction of MHC Class I-Presented Peptides by Incorporating Antigen Processing. *Cell Syst* **11**, 42-48.e47 (2020).

37. Öztürk, H., Özgür, A. & Ozkirimli, E. DeepDTA: deep drug-target binding affinity prediction. *Bioinformatics* **34**, i821–i829 (2018).

38. Wen, Z., He, J., Tao, H. & Huang, S.-Y. PepBDB: a comprehensive structural database of biological peptide-protein interactions. *Bioinformatics* **35**, 175–177 (2019).

39. UniProt Consortium. UniProt: a worldwide hub of protein knowledge. *Nucleic Acids Res.* **47**, D506–D515 (2019).

40. Salentin, S., Schreiber, S., Haupt, V. J., Adasme, M. F. & Schroeder, M. PLIP: fully automated protein-ligand interaction profiler. *Nucleic Acids Res.* **43**, W443–7 (2015).

41. Zhao, M., Lee, W.-P., Garrison, E. P. & Marth, G. T. SSW library: an SIMD Smith-Waterman C/C++ library for use in genomic applications. *PLoS One* **8**, e82138 (2013).

42. Wishart, D. S. et al. DrugBank: a comprehensive resource for in silico drug discovery and exploration. *Nucleic Acids Res.* **34**, D668–72 (2006).

43. Wishart, D. S. et al. DrugBank: a knowledgebase for drugs, drug actions and drug targets. *Nucleic Acids Res.* **36**, D901–6 (2008).

44. Knox, C. et al. DrugBank 3.0: a comprehensive resource for 'omics' research on drugs. *Nucleic Acids Res.* **39**, D1035–41 (2011).

45. Law, V. et al. DrugBank 4.0: shedding new light on drug metabolism. *Nucleic Acids Res.* **42**, D1091–7 (2014).

46. Wishart, D. S. et al. DrugBank 5.0: a major update to the DrugBank database for 2018. *Nucleic Acids Res.* **46**, D1074–D1082 (2018).

47. Kim, S. et al. PubChem 2019 update: improved access to chemical data. *Nucleic Acids Res.* **47**, D1102–D1109 (2019).

48. Hashemifar, S., Neyshabur, B., Khan, A. A. & Xu, J. Predicting protein-protein interactions through sequence-based deep learning. *Bioinformatics* **34**, i802–i810 (2018).

49. Fang, C., Shang, Y. & Xu, D. MUFOLD-SS: New deep inception-inside-inception networks for protein secondary structure prediction. *Proteins* **86**, 592–598 (2018).

50. Chen, M. et al. Multifaceted protein-protein interaction prediction based on Siamese residual RCNN. *Bioinformatics* **35**, i305–i314 (2019).

51. Liu, Y., Wu, M., Miao, C., Zhao, P. & Li, X.-L. Neighborhood Regularized Logistic Matrix Factorization for Drug-Target Interaction Prediction. *PLoS Comput. Biol.* **12**, e1004760 (2016).
