# Supplementary material for: A deep-learning framework for multi-level peptide–protein interaction prediction
Source: Nat Commun. 2021 Sep 15;12:5465. doi: 10.1038/s41467-021-25772-4 (PMC8443569; doi:10.1038/s41467-021-25772-4)
Supplement: Supplementary file 2 — Reporting Summary [file 41467_2021_25772_MOESM2_ESM.pdf]

nature portfolio

Corresponding author(s): Dan Zhao, Jianyang Zeng

Last updated by author(s): Aug 27, 2021

Reporting Summary

Nature Portfolio wishes to improve the reproducibility of the work that we publish. This form provides structure for consistency and transparency in reporting. For further information on Nature Portfolio policies, see our Editorial Policies and the Editorial Policy Checklist.

Statistics

For all statistical analyses, confirm that the following items are present in the figure legend, table legend, main text, or Methods section.

n/a	Confirmed	
☐	☒	The exact sample size (*n*) for each experimental group/condition, given as a discrete number and unit of measurement
☐	☒	A statement on whether measurements were taken from distinct samples or whether the same sample was measured repeatedly
☐	☒	The statistical test(s) used AND whether they are one- or two-sided<br>*Only common tests should be described solely by name; describe more complex techniques in the Methods section.*
☒	☐	A description of all covariates tested
☒	☐	A description of any assumptions or corrections, such as tests of normality and adjustment for multiple comparisons
☐	☒	A full description of the statistical parameters including central tendency (e.g. means) or other basic estimates (e.g. regression coefficient) AND variation (e.g. standard deviation) or associated estimates of uncertainty (e.g. confidence intervals)
☐	☒	For null hypothesis testing, the test statistic (e.g. *F*, *t*, *r*) with confidence intervals, effect sizes, degrees of freedom and *P* value noted<br>*Give P values as exact values whenever suitable.*
☒	☐	For Bayesian analysis, information on the choice of priors and Markov chain Monte Carlo settings
☒	☐	For hierarchical and complex designs, identification of the appropriate level for tests and full reporting of outcomes
☐	☒	Estimates of effect sizes (e.g. Cohen's *d*, Pearson's *r*), indicating how they were calculated

Our web collection on statistics for biologists contains articles on many of the points above.

Software and code

Policy information about availability of computer code

Data collection	python (2.7.12), ssw (1.1), psi-blast (2.9.0+), Striped-Smith-Waterman-Library, PLIP (v1.4.2), SCRATCH-1D (1.2), IUPred2A
Data analysis	The models were implemented using python (2.7.12) with Keras (2.0.8) and Tensorflow (1.2.1). The PDB complexes were visualized using Pymol (2.3.4). All docking experiments were conducted using AutoDock CrankPep v1.0, GalaxyPepDock (http://galaxy.seoklab.org/), CABS-Dock (https://github.com/mchelem/cabsdock-client) and MDockPeP (https://zougrouptoolkit.missouri.edu/mdockpep/). The source codes of CAMP are available on GitHub repository at https://github.com/twopin/CAMP.

For manuscripts utilizing custom algorithms or software that are central to the research but not yet described in published literature, software must be made available to editors and reviewers. We strongly encourage code deposition in a community repository (e.g. GitHub). See the Nature Portfolio guidelines for submitting code & software for further information.

Data

Policy information about availability of data

All manuscripts must include a data availability statement. This statement should provide the following information, where applicable:

- Accession codes, unique identifiers, or web links for publicly available datasets
- A description of any restrictions on data availability
- For clinical datasets or third party data, please ensure that the statement adheres to our policy

The peptide-protein complex structure data used in this study can be downloaded from the RCSB Protein Data Bank (PDB) database [https://www.rcsb.org/downloads/] and the structural peptide-protein interaction data with annotated binding residue information are available from PepBDB [http://huanglab.phys.hust.edu.cn/pepbdb/db/download/]. The corresponding PDB IDs that we used for training and testing the model can be found in Supplementary Table 12 and 13 in Supplementary Data, respectively. The peptide drug-target interaction data are available from DrugBank [https://go.drugbank.com/releases/

latest]. The sequence data of the peptide drugs on DrugBank are available from PubChem [https://pubchem.ncbi.nlm.nih.gov/]. The corresponding DrugBank IDs that we used can be found in Supplementary Table 14. The protein sequence data used in this study are available from UniProt [https://www.uniprot.org/downloads] and the corresponding UniProt IDs that we used for training and testing can be found in Supplementary Table 15 and 16 in Supplementary Data, respectively. The peptide-PBD (protein binding domain) interaction data are available from [https://github.com/aqlaboratory/hsm]. The affinity data of peptide-protein interactions are available from PDBbind v2019 [http://www.pdbbind.org.cn/] and the corresponding PDB IDs that we used for affinity assessment can be found in Supplementary Table 17 in Supplementary Data. The supplementary test sets are available from LEADS-PEP [https://pubs.acs.org/doi/suppl/10.1021/acs.jcim.9b00905/suppl_file/ci9b00905_si_001.pdf], PPDbench [https://webs.iiitd.edu.in/raghava/ppdbench/dataset.php], PepSet [http://cadd.zju.edu.cn/pepset/], TS251 [https://bitbucket.org/isaakh94/interpep_pipeline/src/master/databases/] and TS125 [https://academic.oup.com/bioinformatics/article/34/3/477/4237510#supplementary-data], respectively.

Field-specific reporting

Please select the one below that is the best fit for your research. If you are not sure, read the appropriate sections before making your selection.

☒ Life sciences ☐ Behavioural & social sciences ☐ Ecological, evolutionary & environmental sciences

For a reference copy of the document with all sections, see nature.com/documents/nr-reporting-summary-flat.pdf

Life sciences study design

All studies must disclose on these points even when the disclosure is negative.

Sample size	No sample size calculation was performed. We collected 7,417 samples to construct our benchmark dataset to train and validate our model. We also collected 379 samples to construct an independent test set. The consistent performance through different datasets demonstrates such sample sizes are sufficient.
Data exclusions	When constructing the benchmark dataset, we excluded a specific class of antigen peptides binding to the major histocompatibility complex (MHC) molecules, since they possess specific binding mechanisms that may not be easily generalized to common peptide-protein interactions. In addition, to maintain the high quality of the constructed dataset, we excluded those pairs that contained peptide sequences with more than 20% unknown or non-standard amino acids, or protein sequences that were longer than 5,000 amino acids as (this threshold could cover more than 99% of the protein sequences).
Replication	For model training, we conducted n-fold cross validation (n=5 and n=9), where we independently replicated the training and validating processes for n times. In addition, we reproduced the performance of our model using an independent test dataset described in the paper.
Randomization	The data we used were originated from experiments that were usually randomly allocated since we first randomly shuffled all samples in our dataset after data processing procedure. For model training, we split the training set and testing set using cross-validation strategy. In particular, for the binary prediction task, we used five-fold cross-validation for the "random-split setting". That is, we randomly partitioned the dataset into five folds, and used one of them as the validation set. For the "novel protein setting" and the "novel peptide setting", we conducted five-fold cross-validation on the sequence clusters instead of directly splitting the sequences of proteins or peptides. Here, the proportion of validation set was approximately 20%, since the data amount within each cluster was not always evenly distributed. For the "novel pair setting", we conducted cross-validation on both protein and peptide clusters. In particular, we first split protein clusters into three grids, and then within each grid, we further split the peptide clusters into three grids. In this way, we divided the dataset into nine grids for a nine-fold cross-validation procedure. We chose the data of a single grid as a validation set and the remaining four grids that did not have any overlapped protein or peptide cluster as the training set. Finally, within each fold or each cluster, samples were shuffled again to avoid bias.
Blinding	The investigators were blinded to group allocation.

Reporting for specific materials, systems and methods

We require information from authors about some types of materials, experimental systems and methods used in many studies. Here, indicate whether each material, system or method listed is relevant to your study. If you are not sure if a list item applies to your research, read the appropriate section before selecting a response.

Materials & experimental systems

n/a	Involved in the study
☒	☐ Antibodies
☒	☐ Eukaryotic cell lines
☒	☐ Palaeontology and archaeology
☒	☐ Animals and other organisms
☒	☐ Human research participants
☒	☐ Clinical data
☒	☐ Dual use research of concern

Methods

n/a	Involved in the study
☒	☐ ChIP-seq
☒	☐ Flow cytometry
☒	☐ MRI-based neuroimaging
